# Alternate wiring of a *KNOXI* genetic network underlies differences in leaf development of *A. thaliana* and *C. hirsuta*

**DOI:** 10.1101/gad.269050.115

**Published:** 2015-11-15

**Authors:** Madlen I. Rast-Somssich, Suvi Broholm, Huw Jenkins, Claudia Canales, Daniela Vlad, Michiel Kwantes, Gemma Bilsborough, Raffaele Dello Ioio, Rob M. Ewing, Patrick Laufs, Peter Huijser, Carolyn Ohno, Marcus G. Heisler, Angela Hay, Miltos Tsiantis

**Affiliations:** 1 Department of Comparative Development and Genetics, Max Planck Institute for Plant Breeding Research, 50829 Cologne, Germany;; 2 Department of Plant Sciences, University of Oxford, Oxford OX1 3BR, United Kingdom;; 3 Dipartimento di Biologia e Biotecnologie, Università di Roma, Sapienza, 70-00185 Rome, Italy;; 4 Centre for Biological Sciences, University of Southampton, Southampton SO17 1BJ, United Kingdom;; 5 Institut Jean-Pierre Bourgin, UMR1318, Institut National de la Recherche Agronomique (INRA)-Institut des Sciences et Industries du Vivant et de l'Environment (AgroParisTech), INRA Centre de Versailles-Grignon, 78026 Versailles Cedex 69117, France;; 6 Developmental Biology Unit, European Molecular Biology Laboratory, Heidelberg, Germany

**Keywords:** *KNOXI genes*, regulatory evolution, *CUP-SHAPED COTYLEDON*, *Cardamine hirsuta*, pleiotropy, compound leaf

## Abstract

Two interrelated problems in biology are understanding the regulatory logic and predictability of morphological evolution. Here, we studied these problems by comparing *Arabidopsis thaliana*, which has simple leaves, and its relative, *Cardamine hirsuta*, which has dissected leaves comprising leaflets. By transferring genes between the two species, we provide evidence for an inverse relationship between the pleiotropy of *SHOOTMERISTEMLESS* (*STM*) and *BREVIPEDICELLUS* (*BP*) homeobox genes and their ability to modify leaf form. We further show that *cis*-regulatory divergence of *BP* results in two alternative configurations of the genetic networks controlling leaf development. In *C. hirsuta*, *ChBP* is repressed by the microRNA164A (MIR164A)/*ChCUP-SHAPED COTYLEDON* (*ChCUC*) module and *ChASYMMETRIC LEAVES1* (*ChAS1*), thus creating cross-talk between MIR164A/CUC and AS1 that does not occur in *A. thaliana.* These different genetic architectures lead to divergent interactions of network components and growth regulation in each species. We suggest that certain regulatory genes with low pleiotropy are predisposed to readily integrate into or disengage from conserved genetic networks influencing organ geometry, thus rapidly altering their properties and contributing to morphological divergence.

## Abstract (precis)

In this study, Rast-Somssich et al. investigated morphological differences between *C. hirsuta*, which has complex leaves with leaflets, and its relative, *A. thaliana*, which has simple leaves. By transferring single genes from one species into another under their endogenous regulatory elements, the authors show that leaf form can be modified in the recipient species, extending our knowledge of how paralogous genes are regulated in a complex eukaryote.

One approach to understand the genetic basis for evolutionary change is to identify genes that underlie morphological diversity and investigate how those evolved and how their diversification influenced morphogenesis. In this context, there is considerable interest in determining whether genes or genetic changes underlying morphological differences between species share unifying features, which would indicate that evolution is, to an extent, predictable (Williams et al. 2008; Gompel and Prud'homme 2009; Stern and Orgogozo 2009; Chan et al. 2010; Prud'homme et al. 2011). Current evidence suggests that morphological evolution often results from changes in gene expression of key developmental regulators. Such mutations facilitate morphological change while minimizing the potentially adverse effects of pleiotropy; i.e., the phenomenon by which a single gene influences multiple traits. In this way, regulatory evolution, constrained by pleiotropy, can drive morphological change in specific traits without reducing organismal fitness (Williams et al. 2008; Stern and Orgogozo 2009; Chan et al. 2010; Prud'homme et al. 2011). However, the precise influence of pleiotropy in determining the relative evolutionary potential of different genes remains unclear. Paralogous genes with overlapping functions but different levels of pleiotropy offer an attractive opportunity to investigate this problem. If the evolutionary potential of such genes is highly constrained by their pleiotropy, then we expect the more pleiotropic gene to incur a higher fitness penalty when diversifying. Consequently, we expect this gene to evolve variants that make only modest contributions to trait diversity. In contrast to this, the less pleiotropic paralog with comparable developmental function would be less constrained, and we expect this gene to evolve variants that make a greater contribution to trait diversification.

It is clear that regulatory divergence supports trait evolution and can contribute to the assembly of new genetic modules influencing morphology (Raff 1996; Doebley and Lukens 1998; Carroll 2005; Gompel et al. 2005; Arnoult et al. 2013; Rebeiz et al. 2015). However, the specific impact of diversification at the individual gene level on genetic network architecture remains poorly understood. One well-established possibility is module reuse, whereby broadly conserved genetic interactions are redeployed in space and/or time due to altered gene expression, resulting in morphological diversity (Arthur 2002; Carroll 2008; Mallarino and Abzhanov 2012). Alternatively, the modified expression of a developmental regulator could result in a more radical reorganization of genetic networks through novel genetic interactions, thus amplifying the potential for regulatory changes at a single locus to generate morphological diversity. This possibility remains underexplored owing to the relative paucity of comparative experimental systems that allow developmentally fine-grained investigation into how species-specific genetic variants cause phenotypic diversity.

Leaves of seed plants provide attractive opportunities to study these problems because they show a tremendous degree of heritable, morphological variation (Bar and Ori 2014, 2015). Leaf shapes can be classified as simple (if the blade is entire, as in the model species *Arabidopsis thaliana*) or dissected, also referred to as compound (if the blade is divided into distinct leaflets, as in *Cardamine hirsuta*) (Efroni et al. 2010). Leaves initiate as entire structures at the flanks of the pluripotent shoot apical meristem (SAM), but, in some species, elaboration of novel axes of cell proliferation results in leaflet formation (Ori et al. 2007; Barkoulas et al. 2008). Additionally, the margins of both simple and compound leaves can elaborate less-pronounced incisions, referred to as serrations or lobes depending on their depth (Fig. 1A; Blein et al. 2008; Hasson et al. 2011; Rubio-Somoza et al. 2014).

**Figure 1.**
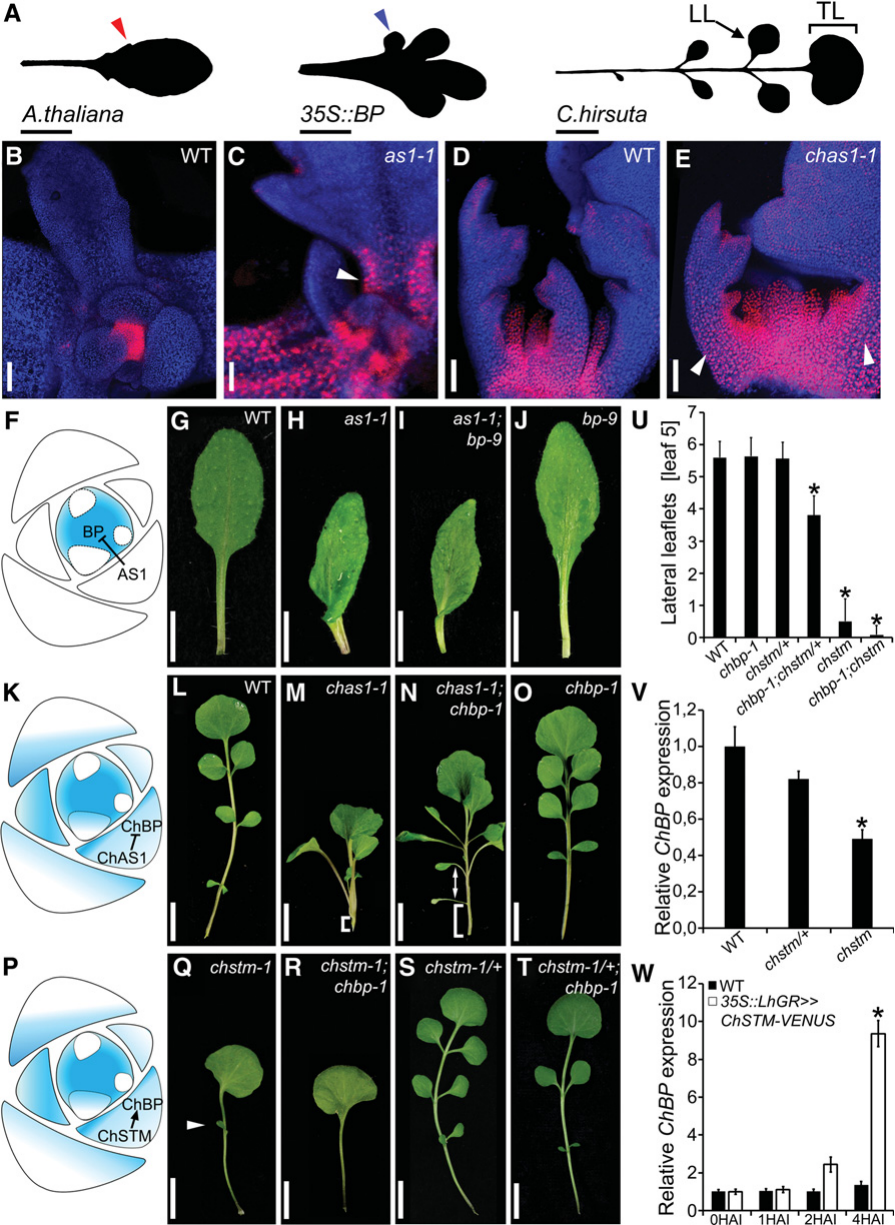
*BREVIPEDICELLUS (BP)* repression by AS1 is conserved between *A. thaliana* and *C. hirsuta* but has different phenotypic significance in the two species. (*A*) Leaf 5 silhouettes of *A. thaliana* wild type with marginal serrations (red arrowhead); plants expressing *BP* under the *35S* promoter, causing the formation of marginal lobes (blue arrowhead); and *C. hirsuta* wild type with lateral (LL) and terminal (TL) leaflets. (*B*–*E*) *BP::VENUS* (*B*,*C*) and *ChBP::VENUS* (*D*,*E*) expression (red) combined with chlorophyll autofluorescence (blue) in *A. thaliana* (*B*,*C*) and *C. hirsuta* (*D*,*E*) wild-type and *as1-1* mutant plants. (*C*,*E*) Note the broadened *BP/ChBP* expression in *as1-1* and *chas1-1* mutants relative to the respective wild type (arrowheads). (*F*) Cartoon of an *A. thaliana* shoot apex: AS1 restricts *BP* expression from the leaf primordia. (*G*–*J*) Leaf 5 of *A. thaliana* wild type (*G*) and a*s1-1* (*H*), *as1-1;bp-9* (*I*), and *bp-9* (*J*) mutants. (*K*) Cartoon of a *C. hirsuta* shoot apex: Both *ChAS1* and *ChBP* are expressed in leaves of *C. hirsuta.* Nevertheless, the repressive interaction is conserved. (*L*–*O*) Leaf 5 of *C. hirsuta* wild type (*L*) and *chas1-1* (*M*), *chas1-1;chbp-1* (*N*), and *chbp-1* (*O*) mutants. Note the suppression of petiole growth arrest (bracket in *M*,*N*) and leaflet positioning defects along the proximodistal axis (arrow in *N*) in *chas1-1;chbp-1* compared with *chas1-1* mutant leaves. (*P*) Cartoon of a *C. hirsuta* shoot apex: *ChSTM* promotes *ChBP* expression during leaflet development. (*Q*–*T*) Leaf 5 of *C. hirsuta chstm-1* (*Q*; arrowhead indicates a rare leaflet), *chstm-1;chbp-1* (*R*), *chstm-1*/+ (*S*), and *chstm-1/+;chbp-1* (*T*) mutants. (*U*) Quantification of lateral leaflets on leaf 5 of plants with the indicated genotype. *n* ≥ 25. (*V*,*W*) *ChBP* transcript levels in *C. hirsuta chstm-1*/+ and *chstm-1* mutants (*V*; 14 d after germination [14DAG]; *n* = 3) and upon induction of *ChSTM* misexpression with 10 mM DEX in *35S::LhGR>>ChSTM-VENUS C. hirsuta* seedlings (*W*; 14DAG; *n* = 3). Error bars in *U*–*W* indicate standard deviation. (HAI) Hours after induction; (asterisk) statistically significant difference from wild-type (*U*,*V*) or uninduced (*W*) samples (*P* ≤ 0.05, Student's *t*-test).

Regulators of leaflet development have been identified in several taxa, but the genetic basis for species-specific leaflet formation remains poorly understood (Blein et al. 2008, 2010; Ben-Gera and Ori 2012; Bar and Ori 2014, 2015). So far, genetic variation in two pathways has been causally connected to differences between simple and dissected leaves. The first involves local growth restriction that promotes leaflet separation and requires the *REDUCED COMPLEXITY* (*RCO*) HD-ZIP I gene, which was discovered in *C. hirsuta* (Vlad et al. 2014) *RCO* evolved through duplication in the Brassicaceae family, and its species-specific activity in leaf diversity results from its unique expression pattern at the base of initiating leaflets. Conversely, loss of *RCO* from the *A. thaliana* genome contributed to leaf simplification in this species (Sicard et al. 2014; Vlad et al. 2014). The second and more extensively studied pathway involves differential expression of class I KNOTTED1-LIKE HOMEOBOX (KNOXI) homeodomain proteins (Hareven et al. 1996; Bharathan et al. 2002; Hay and Tsiantis 2006; Blein et al. 2008; Shani et al. 2009; Furumizu et al. 2015). In most simple-leafed species, including *A. thaliana*, KNOXI proteins are confined to the meristem, where they prevent differentiation (Jackson et al. 1994; Lincoln et al. 1994; Smith et al. 1995; Long et al. 1996). Conversely, in many dissected-leafed species, including *C. hirsuta*, KNOXI proteins also accumulate in leaves, where they promote leaflet development (Bharathan et al. 2002; Hay and Tsiantis 2006, 2010). This differential expression of *KNOXI* genes between simple and dissected leaves results from *cis-*regulatory divergence of *KNOXI* loci (Hay and Tsiantis 2006). However, the regulatory logic underlying *KNOXI*-dependent diversification of leaf morphology remains poorly understood. For example, it is untested whether *KNOXI* genes are sufficient to increase the complexity of simple leaves when expressed from their native regulatory sequences that confer expression in dissected leaves. Such experiments are important, as they are the best available test to evaluate the contribution of genes or regulatory sequences to trait diversification between related species (Chan et al. 2010; Arnoult et al. 2013; Stern and Frankel 2013; Vlad et al. 2014; Rebeiz et al. 2015). Moreover, although upstream components of the KNOXI pathway have been identified (Timmermans et al. 1999; Tsiantis et al. 1999; Byrne et al. 2000; Ori et al. 2000; Ge et al. 2014), it is unclear how the correct *KNOXI* expression domain in dissected leaves is precisely delimited.

Here we studied the contribution of *SHOOTMERISTEMLESS* (*STM*) and *BREVIPEDICELLUS* (*BP*)—two redundantly acting, paralogous *KNOXI* genes—to leaf shape diversity between *C. hirsuta* and *A. thaliana.* Using comparative genetics and cross-species gene transfer assays, we show that the less pleiotropic gene, *BP*, has a higher potency to modify leaf form. We found that the *cis*-regulatory properties of *BP* that underlie its species-specific expression also influence its genetic interactions with conserved regulators of leaf development. Specifically, in the *C. hirsuta* leaf, *ChBP* is concurrently regulated by the microRNA164A (MIR164A)/*ChCUP-SHAPED COTYLEDON* (*ChCUC*) module and *ChASYMMETRIC LEAVES1* (*ChAS1*), thus creating a regulatory linkage between MIR164A/CUC/AS1 that does not occur in *A. thaliana* leaves (Ori et al. 2000; Hay and Tsiantis 2006). We show that this particular regulatory architecture creates novel developmental boundaries that influence leaf shape. Our findings illustrate how the *cis*-regulatory properties of an individual gene may have been influenced by gene pleiotropy to create novel regulatory interactions with considerable impact on leaf morphology.

## Results

### Repression of *ChBP* expression in dissected leaves of *C. hirsuta*

We first compared the regulation of *BP* between *A. thaliana* and *C. hirsuta.* In *A. thaliana*, the AS1 MYB protein prevents *BP* transcription in simple leaf primordia (Waites et al. 1998; Byrne et al. 2000, 2002; Ori et al. 2000; Guo et al. 2008). In contrast, *ChBP* is transcribed in dissected *C. hirsuta* leaves despite being negatively regulated by ChAS1 (Hay and Tsiantis 2006). To analyze *BP* and *ChBP* expression at cellular resolution, we constructed fluorescent reporter gene fusions in both species. We observed ectopic expression of both *BP::VENUS* and *ChBP::VENUS* in *as1* mutant leaves of *A. thaliana* and *C. hirsuta*, respectively (Fig. 1B–E, arrowheads). Moreover, we found that both reporter genes showed broadened and elevated expression in *as1* leaves of *A. thaliana*, suggesting that their divergent *cis*-regulatory properties do not affect their negative regulation by AS1 (Supplemental Fig. S1). Thus, the AS1/BP interaction is conserved but has different developmental significance in the two species. In *A. thaliana*, AS1 excludes *BP* transcription from leaves to safeguard leaf development from inappropriate expression of a meristem gene (Ori et al. 2000; Byrne et al. 2002). In *C. hirsuta, ChBP* is expressed in leaves due to its *cis*-regulatory properties, and ChAS1 defines its expression pattern and dose (Fig. 1F,K).

To understand the consequences of this differential deployment of the AS1/*BP* module for leaf development, we compared the significance of this repressive interaction for morphology in the two species. In *A. thaliana*, the leaf phenotypes of *as1;bp* double mutants do not deviate appreciably from *as1* single mutants (Fig. 1G–J) because other genes, including *BP* paralogs, contribute to the *as1* mutant phenotype (Byrne et al. 2000; Ori et al. 2000; Ikezaki et al. 2010). We reasoned that *chbp* loss-of-function alleles might condition stronger suppression of the *chas1* phenotype because both genes are active in the leaf of *C. hirsuta.* To test this hypothesis in an unbiased fashion, we conducted a genetic screen for suppressors of *chas1*, from which we recovered a loss-of-function *chbp* allele (Fig. 1L–O; Supplemental Fig. S2A,B). Quantification of the *chas1;chbp* double-mutant phenotype revealed that repression of growth along the proximodistal axis of *chas1* leaves is strongly *ChBP*-dependent, indicating that ectopic *ChBP* expression contributes to the *chas1* mutant leaf phenotype. Conversely, reanalysis of *as1;bp* double mutants in *A. thaliana* Col-0 showed only very subtle effects on proximodistal leaf growth, as did *as1*;*bp* double mutants in the *A. thaliana* L*er* ecotype, confirming that this double-mutant phenotype is not allele- or background-specific (Table 1).

**Table 1.**
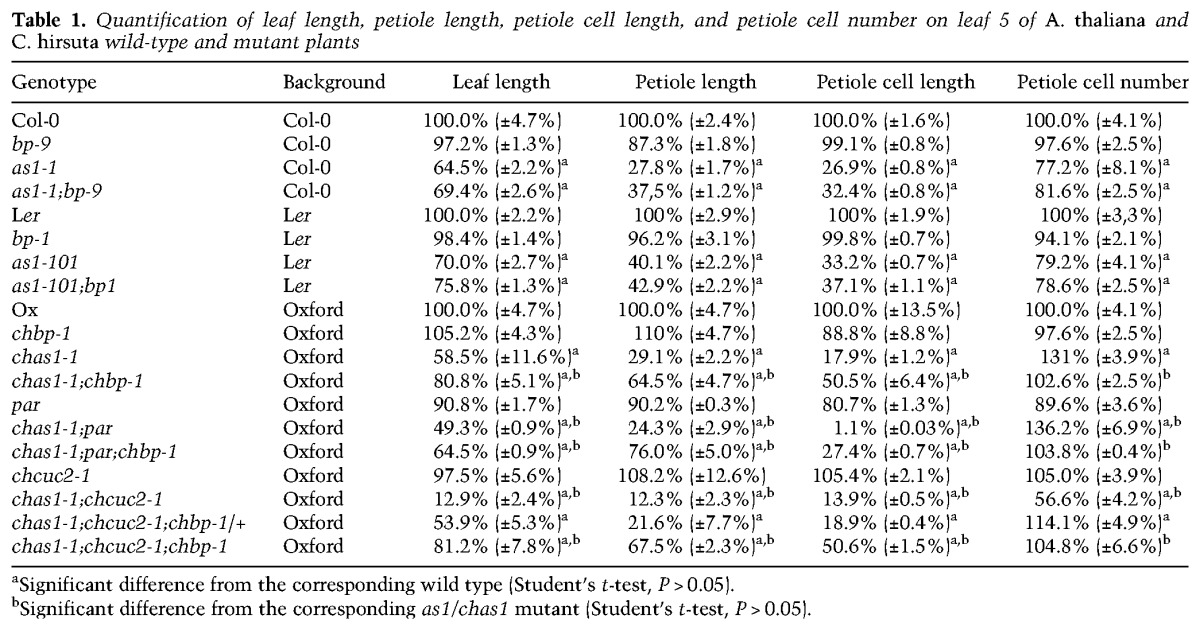
Quantification of leaf length, petiole length, petiole cell length, and petiole cell number on leaf 5 of *A. thaliana* and *C. hirsuta* wild-type and mutant plants

We next investigated whether the reduced length of *chas1* and *as1* mutant leaves reflects a reduction in cell proliferation or cell expansion. To this end, we analyzed epidermal cell size and number along the adaxial leaf surface. We found that leaf epidermal cells of *chas1* and *as1* mutants fail to elongate, particularly in the leaf petiole. This defect is strongly suppressed in *C. hirsuta chas1;chbp* but not in *A. thaliana as1;bp* double mutants (Table 1). Thus, *ChAS1*-dependent regulation of *ChBP* expression is required to define the correct timing of leaf cell elongation and differentiation and, consequently, growth along the proximodistal axis. These observations indicate that *ChBP* accounts for a considerable proportion of *ChAS1* function in *C. hirsuta* leaves, suggesting that this interaction is more important for leaf growth and development in *C. hirsuta* than in *A. thaliana*.

### *ChBP* and *ChSTM* act redundantly to promote leaflet formation

To further investigate the function of the *ChAS1/ChBP* module in *C. hirsuta* leaf development, we evaluated the role of *ChBP* in leaflet formation. *chbp* single mutants do not show leaflet number or positioning defects (Fig. 1O), indicating that other genes likely act redundantly with *ChBP* in *C. hirsuta* leaflet formation. Previous work indicated that *ChSTM* is required for leaflet formation and that *cis*-regulatory differences contribute to *ChSTM* expression in *C. hirsuta* leaves and exclusion of *STM* expression from *A. thaliana* leaves (Hay and Tsiantis 2006). Consequently, we postulated that *ChBP* and *ChSTM* might act redundantly to promote leaflet production. This would be in line with redundant functions of the related genes *BP* and *STM* in *A. thaliana* SAM maintenance and *Rough sheath1* and *Knotted1* in maize shoot development (Byrne et al. 2002; Bolduc et al. 2014). We tested this hypothesis with a hypomorphic *chstm* allele that we isolated from a genetic screen for mutants with reduced leaflet formation. Both the homozygous *chstm* single mutant and the *chstm;chbp* double mutant had meristem defects but retained some ability to make leaves (Supplemental Fig. S2D–G). However, the frequency of leaf formation was significantly lower in *chstm-1;chbp* than in *chstm* mutants. Furthermore, *chstm*/+ had a dose-dependent effect on leaflet formation in a *chbp* background, as *chstm/+;chbp* plants produced significantly fewer lateral leaflets than wild type without influencing SAM function (Fig. 1P–U; Supplemental Fig. S2H–J,L). Thus, *ChBP* acts redundantly with *ChSTM* to promote meristem function and leaflet formation. The basis of this redundancy is likely multifaceted, as *ChSTM* is a positive regulator of *ChBP* expression in leaves (Fig. 1V,W; Supplemental Fig. S2M), and previous work indicated that STM and BP physically interact (Smith and Hake 2003).

Although *chstm* mutants show leaflet defects, *chbp* mutants only show such defects in a *chstm*/+ background. This observation indicates that while *ChSTM* and *ChBP* act redundantly to promote leaflet formation, there is a stricter requirement for *ChSTM* function. *STM* has a broader role than *BP* throughout development in both *A. thaliana* and *C. hirsuta*, as seen from the pronounced SAM and organogenic defects and infertility found in *stm* but not *bp* mutants (Supplemental Fig. S2B–E; Endrizzi et al. 1996; Byrne et al. 2002; Hay et al. 2002; Smith and Hake 2003). In conclusion, the two *KNOXI* genes *ChSTM* and *ChBP* redundantly promote leaflet development, but *ChSTM* has a more central role in the process and more pleiotropic effects during development.

### The ability of *ChBP* and *ChSTM* to alter *A. thaliana* leaf shape: evidence for a tradeoff between gene pleiotropy and potency

**Figure 2.**
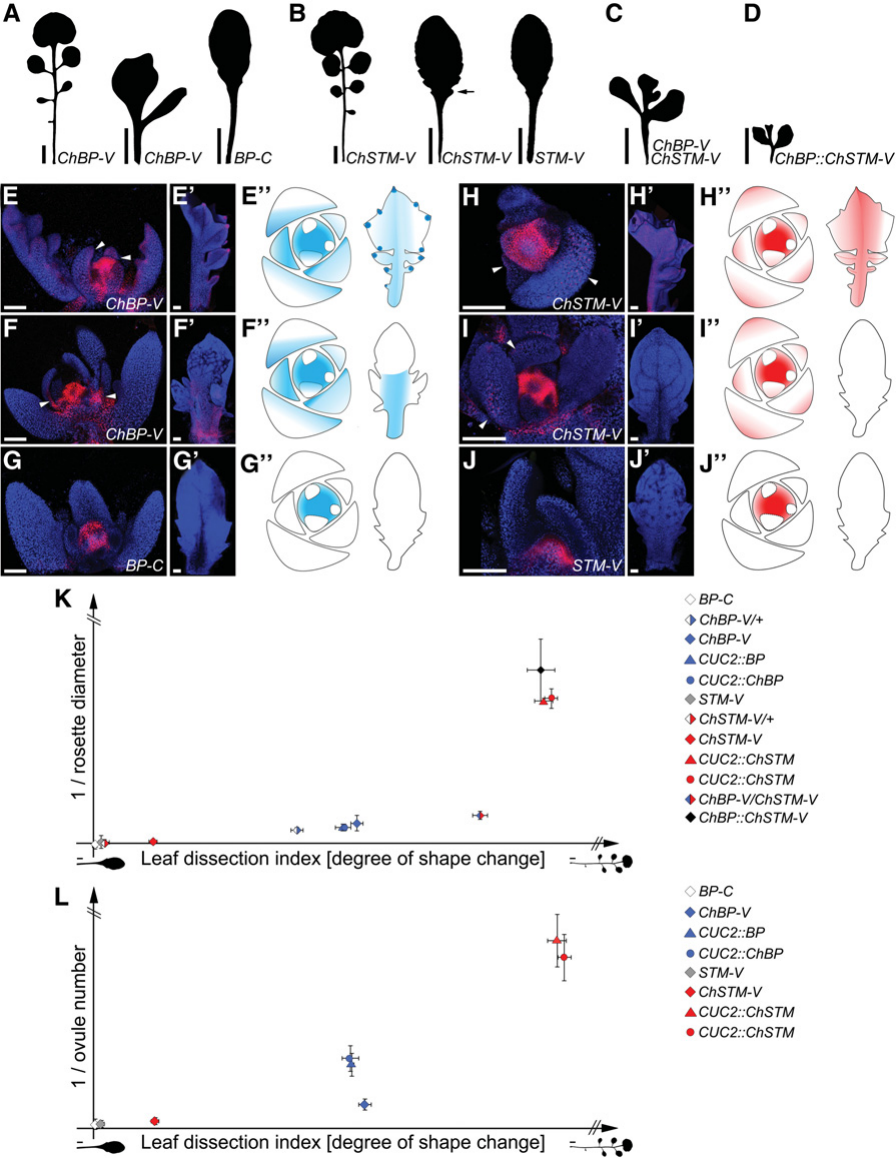
*ChBP* is less pleiotropic but more potent in altering *A. thaliana* leaf shape than *ChSTM*. (*A*–*D*) Leaf 5 silhouettes of transgenic *C. hirsuta* (first silhouette in *A*,*B*) and *A. thaliana* (second and third silhouettes in *A*,*B*; *C*,–*D*) lines expressing *ChBP::ChBP-VENUS* (*ChBP-V*), *BP::BP-CFP* (*BP-C*), *ChSTM::ChSTM-VENUS* (*ChSTM-V*), *STM::STM-VENUS* (*STM-V*), both *ChBP-V* and *ChSTM-V* (*C*), or *ChBP::ChSTM-VENUS* (*ChBP-ChSTM-V*) (*D*). (*E*–*J*′′) Maximum intensity projections of confocal stacks showing reporter gene expression (red) combined with chlorophyll autofluorescence (blue) (*E*–*J*′) and cartoons of a shoot apex and a leaf (500 µm) summarizing the observed expression in transgenic *C. hirsuta* and *A. thaliana* lines (*E*′′–*J*′′). *ChBP-V* expression in *C. hirsuta* (*E*–*E*′′) and *A. thaliana* (*F*–*F*′′). (*G*–*G*′′) *BP-C* expression in *A. thaliana. ChSTM-V* expression in *C. hirsuta* (*H*–*H*′′) and *A. thaliana* (*I*–*I*′′). (*J*–*J*′′) *STM-V* expression in *A. thaliana.* Expression of *ChBP-V* and *ChSTM-V* is detectable in the *A. thaliana* SAM and leaves (arrowheads in *E*,*F*,*H*,*I* indicate leaf-specific expression), but *ChSTM-V* expression is not sustained after the leaf reaches a size of 400 µm (*I*′). (*K,L*) Diagrams depicting the degree of leaf shape change (calculated as leaf dissection index) versus the reduction in rosette diameter (*K*) or ovule number (*L*) caused by each transgene (Supplemental Fig. S3P–R). Genotypes are indicated in the key. To evaluate the effect of transgene zygosity in *ChBP-V/ChSTM-V* plants, the *ChBP-V* and *ChSTM-V* homozygous lines were backcrossed to wild type (*ChBP-V*/+ and *ChSTM-V*/+) and analyzed in the F1. Bars: *A*–*D*, 1 cm; *E*–*J*′, 100 µm.

We next investigated how the difference in pleiotropy of *ChBP* and *ChSTM* associates with the sufficiency of each of the two loci to alter leaf shape from simple to more complex. To this end, we introduced two transgenes, *ChBP::ChBP-VENUS* (*ChBP-V*) and *ChSTM::ChSTM-VENUS* (*ChSTM-V*), into wild-type *A. thaliana* plants and evaluated the relative potency of each gene to shift the morphology of the recipient species (*A. thaliana* with simple leaves) to that of the species of origin (*C. hirsuta* with dissected leaves). Because both transgenes complemented their respective loss-of-function phenotypes in *C. hirsuta* (Supplemental Fig. S2K,N–R), we reasoned that they drive *KNOXI* expression in *A. thaliana* from a *cis*-regulatory context faithful to their native one in *C. hirsuta.* Morphological analysis of the resulting transgenic lines demonstrated that expression of both *ChBP-V* and *ChSTM-V* was sufficient to shift the *A. thaliana* simple leaf to a more complex form. However, *ChBP-V* was considerably more potent than *ChSTM-V* in altering *A. thaliana* leaf morphology despite the more stringent requirement for *ChSTM* in *C. hirsuta* leaflet development (Fig. 2A,B).

We investigated whether the differences in potency to alter leaf shape between *ChBP-V* and *ChSTM-V* could be explained by differences in expression of the two transgenes. We found *ChBP-V* expression in the SAM and leaves of both *C. hirsuta* and *A. thaliana* (Fig. 2E–E′′,F–F′′), while an *A. thaliana BP::BP-CFP* (*BP-C*) reporter generated expression only in the SAM (Fig. 2G–G′′). Analysis of *ChSTM-V* showed expression in the SAM and developing leaf primordia in both species (Fig. 2H–H′′,I–I′′), whereas the *A. thaliana* promoter drove expression only in the SAM (Fig. 2J–J′′). However, in contrast to *C. hirsuta*, *ChSTM-V* expression in *A. thaliana* was not detectable after leaf primordia grew beyond a size of 400 µm (Fig. 2I′). This observation could be explained by factors active in *C. hirsuta* but not *A. thaliana* leaves to maintain *ChSTM-V* expression or repressors present only in *A. thaliana* that down-regulate *ChSTM-V* expression at later stages of leaf development. This premature cessation of *ChSTM-V* expression likely explains its weaker effect on *A. thaliana* leaf development. In contrast to *ChSTM-V*, the regulatory information in *ChBP-V* is sufficient to drive sustained expression throughout leaf development in a pattern similar to that observed at the adaxial site of *C. hirsuta* leaves and endow *A. thaliana* with a complex leaf partly resembling that of the donor species *C. hirsuta.* In conclusion, *ChSTM* and *ChBP* act redundantly to promote leaflet formation in *C. hirsuta*, and it is the least pleiotropic of these genes, *ChBP*, that is more potent in changing *A. thaliana* leaf form in a cross-species gene transfer assay.

These results suggest an inverse relationship between the pleiotropy of *ChSTM* and *ChBP* and the ability of these genomic loci to alter *A. thaliana* leaf shape, which is largely determined by their expression properties. To compare the ability of each protein to increase leaf complexity, we expressed *STM*/*ChSTM* and *BP/ChBP* in the restricted *CUC2* marginal domain of *A. thaliana* lateral organs. Each transgene considerably increased leaf complexity and caused the formation of leaflet-like structures, confirming that the subtle phenotype of the *ChSTM-V* construct in *A. thaliana* is not a result of reduced protein function (Supplemental Fig. S3A–K). We revealed an enhancement of the *ChBP-V* leaf phenotype in *A. thaliana* by additional expression of the *ChSTM-V* transgene, which further indicates that both genes have independent effects on leaf morphology, albeit with different severities (Fig. 2C; Supplemental Fig. S3L). To directly test the contribution of regulatory sequences to the potency of these *KNOXI* genes to influence leaf development, we expressed *ChSTM-VENUS* from the *ChBP* promoter. We found that this transgene considerably increased *A. thaliana* leaf complexity compared with *ChSTM-V* but also severely perturbed plant growth (Fig. 2D; Supplemental Fig. S3M). In fact, the change in leaf shape in the *ChBP::ChSTM-V* and *CUC2::STM/ChSTM* lines is associated with severely arrested leaf and plant growth, but this is not the case in *ChBP-V* and *CUC2::ChBP* lines (Supplemental Fig. S3N–Q). Thus, ChSTM/STM-mediated changes in leaf geometry are associated with a higher penalty for the normal progression of plant development. This observation is visualized in Figure 2K, where the change in *A. thaliana* leaf shape in response to each *BP* or *STM* transgene is quantified relative to the arrest of plant rosette growth caused by this transgene. Comparable results were obtained by quantifying ovule number in selected genotypes (Fig. 2L; Supplemental Fig. S3R). In conclusion, comparative consideration of the consequences of STM/ChSTM and BP/ChBP gain of function shows that the increased leaf complexity caused by STM/ChSTM is concomitant with broader developmental defects. Together with the more pervasive defects caused by loss of STM/ChSTM compared with BP/ChBP, our results indicate that *STM* orthologs are more pleiotropic than their *BP* paralogs. We propose that lower pleiotropy may have allowed *ChBP* to evolve a higher level of evolutionarily relevant *cis*-regulatory activity in leaves. This activity is revealed by the *ChBP* locus being more potent than *ChSTM* in modifying *A. thaliana* leaf form toward a complex shape in our cross-species gene transfer experiments. Consistent with the view that *STM* evolved in a more constrained fashion than *BP*, crucifer *STM* sequences are more conserved than their *BP* counterparts in both coding and noncoding regions (Supplemental Fig. S3S–X; Aguilar-Martinez et al. 2015).

### Dissection of the genetic networks influencing *ChBP* expression

**Figure 3.**
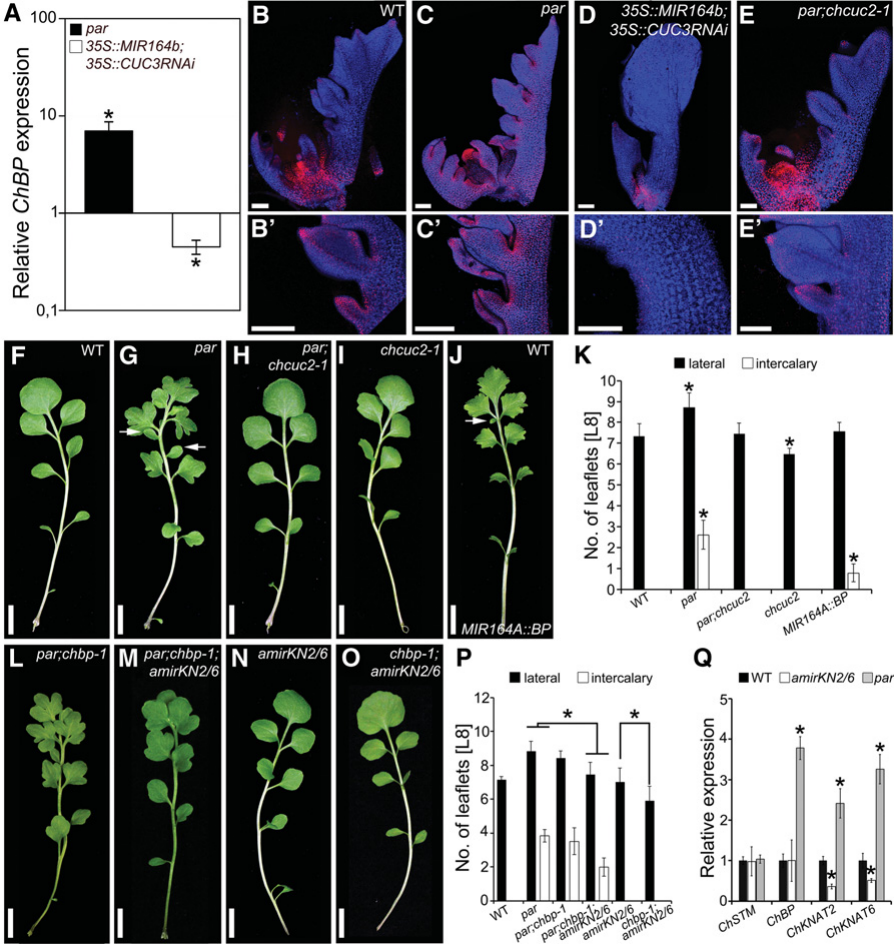
Ectopic *ChBP* expression contributes to the *par* mutant phenotype. (*A*) *ChBP* transcript level in *par* and *35S::MIR164b;CUC3RNAi* (Nikovics et al. 2006) relative to wild-type (set as 1) leaves. (*B*–*E*′) *ChBP::VENUS* expression (red) combined with chlorophyll autofluorescence (blue) in *C. hirsuta* wild-type (*B*,*B*′), *par* (*C*,*C*′), *35S::MIR164B;CUC3RNAi* (*D*,*D*′), and *par;chcuc2-1* (*E*,*E*′) leaves. Shown are maximum intensity projections of confocal stacks. (*F*–*J*) Leaf 8 of *C. hirsuta* wild-type (*F*), *par* (*G*), *par;chcuc2-1* (*H*), and *chcuc2-1* (*I*) plants and transgenic plants expressing *MIR164A::BP* (*J*). (*K*) Quantification of lateral and intercalary leaflet number on leaf 8 of plants with the indicated genotype. *n* ≥ 25. (*L*–*O*) Leaf 8 of *par;chbp-1* (*L*), *par;chbp-1;amirKN2/6* (*M*), *amirKN2/6* (*N*), and *chbp-1;amirKN2/6* (*O*) plants. (*P*) Quantification of lateral and intercalary leaflet number on leaf 8 of plants with the indicated genotype. *n* ≥ 25. (*Q*) Relative expression of *ChSTM*, *ChBP*, *ChKNAT2*, and *ChKNAT6* in *par* and *amirKN2/6* leaves compared with wild type (*n* = 3). Bars: *B*–*E*′, 100 μm; *F*–*O*, 1 cm. Error bars in *K*, *P*, and *Q* indicate standard deviation. (Arrows) Intercalary leaflets; (asterisks) statistically significant difference from wild-type (*K*,*Q*) or the indicated genotype (*P*) (*P* ≤ 0.05, Student's *t*-test).

Our results show that regulation of *BP* expression may be a central process on which evolution acts to influence leaf morphology. To gain insight into *ChBP* regulation in dissected leaves, we sought to identify additional mutants with increased leaflet number phenotypes conditioned by elevated *ChBP* expression (Hay and Tsiantis 2006). We isolated the *parsley* (*par*) mutant and found broadened *ChBP* expression in leaves, suggesting that *PAR* may influence leaf shape by controlling *ChBP* expression (Fig. 3A–C; Supplemental Fig. S4A–F). Through a map-based cloning approach, we showed that *PAR* corresponds to *ChMIR164A* (Supplemental Fig. S4G). In *Arabidopsis*, *mir164A* influences leaf development via delimiting the expression domain of CUC transcription factors (Nikovics et al. 2006). CUC proteins promote the formation of auxin peaks that underlie formation of serrations and leaflets in the leaf margins of *A. thaliana* and *C. hirsuta* (Hay et al. 2006; Barkoulas et al. 2008; Bilsborough et al. 2011; Rubio-Somoza et al. 2014). Consistent with this, we observed increased *ChCUC2* expression, additional convergence points of the PINFORMED1 (PIN1) auxin efflux carrier, and auxin activity maxima in *par* mutant leaves associated with the position of ectopic intercalary leaflet formation along the rachis (Supplemental Fig. S4H–N). The regulation of *ChCUC2* expression and auxin homeostasis is therefore a conserved function of MIR164A action in *A. thaliana* and *C. hirsuta.* However, *mir164a* mutants of *A. thaliana* do not misexpress *BP* in leaves (Nikovics et al. 2006; Bilsborough et al. 2011). Thus, the regulation of *ChBP* in *C. hirsuta* is likely a species-specific function of the *PAR (ChMIR164A*/*ChCUC)* module. This idea is supported by two further observations: that *ChBP* expression is strongly reduced in a *35S:MIR164B;CUC3RNAi* transgenic line (Fig. 3A,D) where expression of *ChCUC1–3* genes is reduced (Nikovics et al. 2006) and that *ChBP* expression is increased upon induced *CUC2* misexpression (Supplemental Fig. S4O).

The above observations indicate that a key difference between the gene regulatory networks (GRNs) controlling leaf shape in *A. thaliana* and *C. hirsuta* is that *ChBP* is expressed in *C. hirsuta* leaves and regulated by *PAR/ChCUC2*. Three lines of evidence support this view and underscore the significance of *PAR/ChCUC2*-mediated restriction of *ChBP* for leaf morphology. First, through a mutant screen, we isolated a *chcuc2* allele as an extragenic suppressor of *par* that is sufficient to revert the *ChBP* expression and leaf phenotype to a near wild-type pattern (Fig. 3E–I; Supplemental Fig. S4P–U). This indicates that elevated *ChCUC2* expression is a major contributor to both the morphological defects and the elevated/broadened *ChBP* expression seen in *par.* Second, *ChBP* expression in the *MIR164A* domain is sufficient to mimic the leaf lobing and increased lateral and intercalary leaflet numbers found in *par* (Fig. 3J,K). Thus, a *ChCUC-*dependent increase in *ChBP* expression likely contributes to the *par* phenotype. Third, we found that two additional *KNOXI* genes, *ChKNAT2* and *ChKNAT6*, contribute redundantly with *ChBP* to the *par* leaf phenotype. Simultaneous silencing of *KNAT2* and *KNAT6* through an artificial microRNA (*amirKN2/6*) combined with the *chbp* allele resulted in a significant reduction of lateral and intercalary leaflet number compared with *par* single or *par;chbp* double mutants (Fig. 3L,M). Furthermore, these three *KNOXI* genes act redundantly to define leaflet number during wild-type leaflet development (Fig. 3N–P). This is in line with the increased *ChBP, ChKNAT2*, and *ChKNAT6* but not *ChSTM* transcript levels in *par* mutant leaves (Fig. 3Q). In conclusion, the *PAR/ChCUC2* module regulates *ChBP* (and *ChKNAT2/6*) expression during *C. hirsuta* leaf development. Our genetics show that *PAR* acts via *ChCUC2* and that *ChBP*/*ChKNAT2/6* account for a considerable proportion of this activity. This regulation is pivotal for defining leaflet number and position. Nevertheless, leaflets remain lobed in *par;chbp;amirKN2/6* mutants, indicating that other genes downstream from *ChCUC2* are required to fully restore the *par* phenotype (Fig. 3M). Candidate genes include components of the auxin homeostasis machinery that account for the altered distribution of PIN1 and auxin activity in *par* mutants (Supplemental Fig. S3J,K).

### Investigation of genetic interactions within the *ChBP/ChCUC* GRN

**Figure 4.**
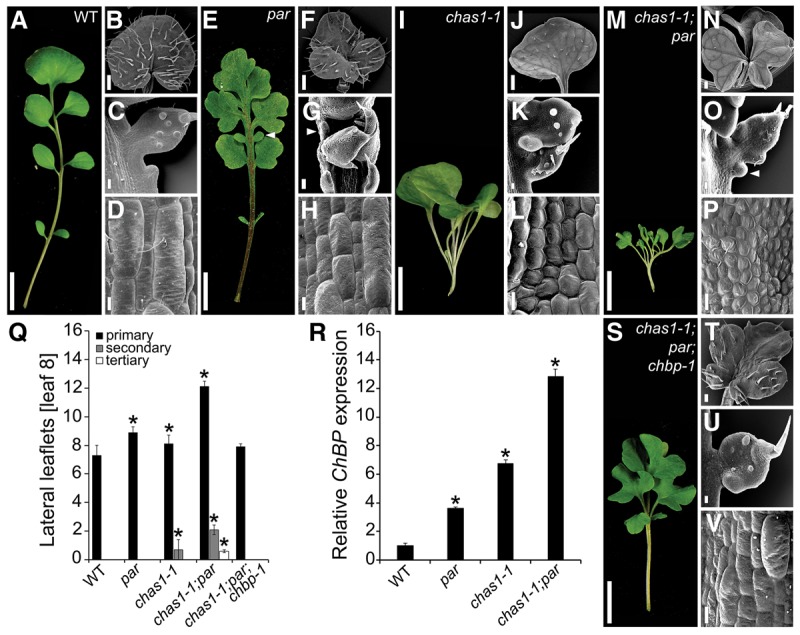
The ChAS1 and *PAR/ChCUC* pathways converge on *ChBP* regulation. (*A*–*P*) *C. hirsuta* wild-type (*A*–*D*), *par* (*E*–*H*), *chas1-1* (*I*–*L*), and *chas1-1;par* (*M*–*P*). For each genotype, rosette leaf 5 (*A*,*E*,*I*,*M*) and scanning electron micrographs of the terminal leaflet (*B*,*F*,*J*,*N*), lateral leaflet (*C*,*G*,*K*,*O*), and epidermal cells on the adaxial surface of the leaf petiole (*D*,*H*,*L*,*P*) are shown. (*Q*) Quantification of lateral leaflet number (primary, secondary, and tertiary) on leaf 8 of plants with the indicated genotype. *n* ≥ 15. (*R*) *ChBP* transcript level in *C. hirsuta* wild-type and *par*, *chas1-1*, and *chas1-1;par*. *n* = 3. (*S*–*V*) Rosette leaf 5 (*S*), terminal leaflet (*T*), lateral leaflet (*U*), and leaf petiole adaxial epidermal cells (*V*) of the *chas1-1;par;chbp-1* mutant. Bars: *A*,*E*,*I*,*M*,*S*, 1 cm; *B*,*F*,*J*,*N*,*T*, 500 µm; *C*,*G*,*K*,*O*,*U*, 100 µm; *D*,*H*,*L*,*P*,*V*, 20 µm. Error bars in *Q* and *R* indicate standard deviation. The asterisks in *Q* and *R* indicate statistically significant difference from wild type (*P* ≤ 0.05, Student's *t*-test).

Our results point to two repressors influencing *ChBP* expression in the *C. hirsuta* leaf: *ChAS1* and *PAR* (*ChMIR164A*), with *PAR* acting via *ChCUC2*. To test how those two repressors interact, we studied *chas1;par* double mutants. We found enhanced leaflet lobing and increased leaflet numbers compared with either single mutant as well as the presence of tertiary leaflets, a phenotype not observed in either single mutant (Fig. 4A–Q). This finding indicates that *ChAS1* and *PAR/ChCUC2* act in parallel pathways to regulate leaflet development and suggests that these two pathways converge on regulation of *ChBP*.

Consistent with this view, the level of *ChBP* expression is substantially higher in *chas1;par* double-mutant than in *chas1* or *par* single-mutant leaves (Fig. 4R). This increase in *ChBP* misexpression also correlates with an enhanced repression of leaf growth along the proximodistal axis of *chas1;par* leaves and is accompanied by a further reduction in epidermal cell length, suggesting a further delay in cell differentiation (Table 1). We found that all phenotypic defects observed in *chas1;par* mutants are at least partially suppressed in *chas1;par;chbp* leaves, indicating that the enhancement of *chas1* by *par* largely depends on *ChBP* (Fig. 4Q–V; Table 1). Thus, *ChBP* emerges as a key convergence point through which the upstream repressors *ChAS1* and *PAR* act. It is noteworthy that the *chas1* phenotype is suppressible by *chbp*, whereas the *par* phenotype is not. This higher contribution of *ChBP* to the *chas1* versus *par* phenotypes may reflect the broader misexpression of *ChBP* in *chas1* than in *par* (Figs. 1D, 3C).

Based on these findings in *C. hirsuta*, we hypothesized that *CUC2* expression in the simple leaves of *A. thaliana* would predispose the leaf GRN to place *BP* under the influence of the *MIR164A/CUC2* module if *BP* is expressed in leaves. We tested this hypothesis by crossing *mir164a-4* and *as1* mutants in *A. thaliana* and found an enhanced leaf phenotype in the double mutant (Supplemental Fig. S5A–H). Furthermore, the ectopic *BP* expression in the leaves of the *as1;mir164a-4* double mutants strictly depends on *CUC2*, as, in *as1;mir164a;cuc2* triple mutants, *BP::GUS* (β*-glucuronidase*) expression is again confined to the SAM. Thus, a single genetic event causing *BP* expression in leaves can in principle be sufficient to allow MIR164A/CUC2-dependent *BP* regulation (Supplemental Fig. S5I–J). In this scenario, a pre-existing module can “capture” a new gene—in this case, *BP*—when its expression diversifies.

**Figure 5.**
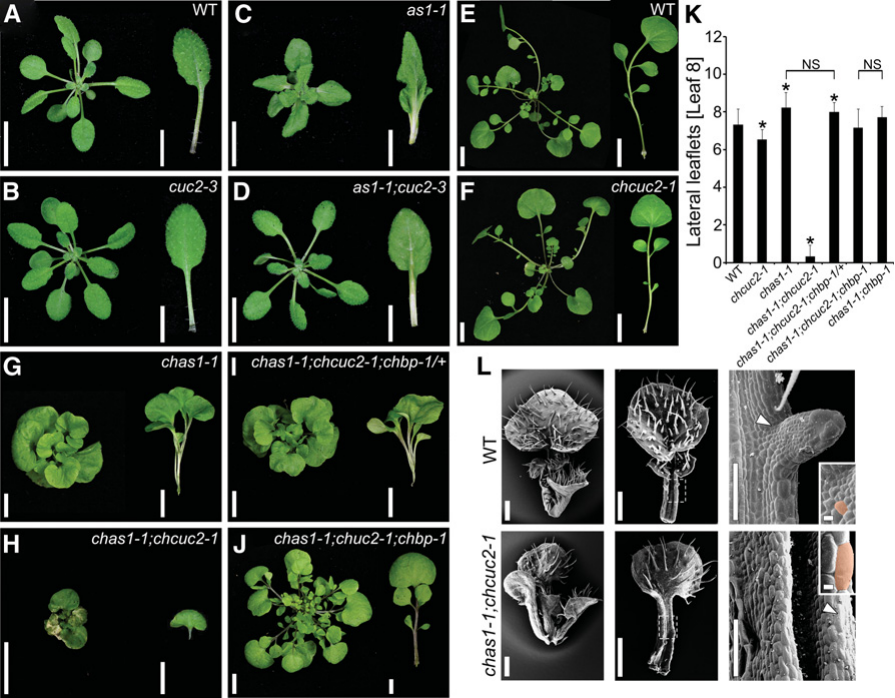
Genetic interactions between *AS1* and *CUC2* in *A. thaliana* and *C. hirsuta.* (*A*–*D*) Rosettes and leaf 5 of *A. thaliana* wild-type (*A*) and *cuc2-3* (*B*), *as1-1* (*C*), and *as1-1;cuc2-3* (*D*) mutant plants. (*E*–*J*) Rosettes and leaf 5 of *C. hirsuta* wild-type (*E*) and *chcuc2-1* (*F*), *chas1-1* (*G*), *chas1-1;chcuc2-1* (*H*), *chas1-1;chcuc2-1;chbp-1*/+ (*I*), and *chas1-1;chcuc2-1;chbp-1* (*J*) mutant plants. (*K*) Quantification of lateral leaflet number on leaf 8 of plants with the indicated genotype. Asterisks indicate significant differences from wild type. *n* ≥ 15. (*L*) Scanning electron micrographs of a vegetative shoot, the fifth developing rosette leaf (1000 µm), and the leaf margin of wild type (*top* panel) and *chas1-1;chcuc2-1* mutants (*bottom* panel). The *insets* show typical cells in the boundary (wild type; arrowhead) or marginal (*as1-1;chcuc2-1*; arrowhead) region, indicated in orange. Bars: *A*–*J*, 1 cm; *L*, 100 µm. Error bars in *K* indicate standard deviation. The asterisks in *K* indicate significant difference from wild type (*P* ≤ 0.05, Student's *t*-test). (NS) No significant difference.

Together, our results provide evidence that, in the *C. hirsuta* leaf, *ChAS1* and *ChCUC2* become interconnected through their combined input on *ChBP* expression. Thus, *ChBP* expression in the leaf creates a node in the *C. hirsuta* leaf GRNs that does not exist in *A. thaliana.* To evaluate the consequences of this alterative GRN organization on genetic interactions between *ChAS1* and *ChCUC2* during leaf development, we studied the *chas1;chcuc2* double-mutant phenotype. In contrast to *A. thaliana*, where *cuc2* suppresses *as1* (Fig. 5A–D; Bilsborough et al. 2011), we observed severely increased growth defects in *C. hirsuta chas1;chcuc2* double mutants that are characterized by a strong reduction in rosette diameter, decreased leaf and petiole length, and a failure to initiate lateral leaflets (Fig. 5E–H). Notably, this genetic interaction between *ChAS1* and *ChCUC2* is *ChBP-*dependent in a dose-sensitive fashion. Specifically, the *chas1;chcuc2;chbp*/+ mutant phenotype was suppressed with respect to *chas1;chcuc2* and resembled *chas1* mutants, and *chas1;chcuc2;chbp* mutants were indistinguishable from *chas1;chbp* mutants (Fig. 5I–K). Thus, the *C. hirsuta* leaf context reveals a combined requirement of *ChAS1* and *ChCUC2* for leaf growth that is species-specific and *ChBP*-dependent.

We found that the reduction in plant size and leaf length in *chas1;chcuc2* double mutants was attributable to a reduction in cell number rather than cell elongation (Table 1), suggesting that ChAS1 and ChCUC2 are together required for cell proliferation in the leaf. It is known that *CUC* genes influence growth in multiple developmental boundaries (Aida et al. 1997; Vroemen et al. 2003; Breuil-Broyer et al. 2004; Wang et al. 2015), while *AS1* and its orthologs regulate growth redundantly with other, partially uncharacterized factors (Waites et al. 1998). Therefore, one possibility is that *ChCUC2* and *ChAS1* act together to regulate *BP* expression and promote formation of boundary domains required for leaflet development and leaf growth. Consistent with this view, the small cells that typically mark the boundary between leaflet and rachis are absent in *chas1;chcuc2* leaves (Fig. 5L, insets).

**Figure 6.**
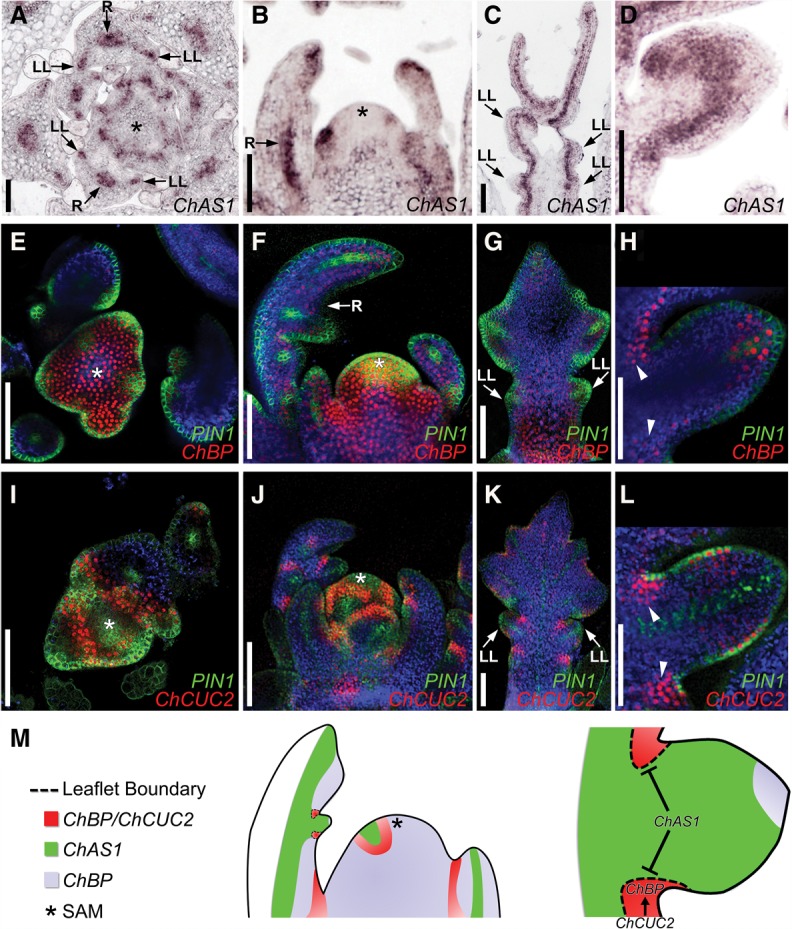
*ChCUC* and *ChAS1* define boundary domains of *ChBP* expression. (*A*–*D*) *ChAS1* expression in the *C. hirsuta* SAM and leaves analyzed by RNA in situ hybridization. *ChBP::VENUS; PIN1::PIN1-GFP* expression (*E*–*H*) and *ChCUC2g-VENUS; PIN1::PIN1-GFP* (*I*–*L*) in the *C. hirsuta* SAM and young leaves. (Red) Venus fluorescence; (green) GFP fluorescence; (blue) chlorophyll autofluorescence. Shown are transverse (*A*,*E*,*I*) and longitudinal (*B*,*F*,*J*) sections through the SAM; leaf 5 at a length of 750 µm (*C*,*G*,*K*); and a close-up of developing lateral leaflets (*D*,*H*,*L*). (*M*) Cartoon summarizing the observed *ChAS1*, *ChBP*, and *ChCUC2* expression patterns. In leaf primordia, *ChBP* is expressed in the adaxial side and at the margin of the rachis (*F*,*G*) as well as at the tip of developing leaflets (*H*). (*H*,*L*) This expression pattern is near complementary to that of *ChAS1* and overlaps with that of *ChCUC2* in the leaf rachis margin at leaflet boundaries (arrowheads). This results in the formation of boundary domains (outlined with dotted lines) that are highly sensitive to *ChBP* dose and required to promote leaf growth. Bars, 100 µm. (R) Leaf rachis; (LL) lateral leaflet. Asterisks mark the SAM.

To further investigate this hypothesis that *ChCUC2* and *ChAS1* may jointly regulate leaflet boundary formation via promoting and repressing the expression of *ChBP*, respectively, we determined the relative expression patterns of *ChAS1* and *ChCUC2* during *C. hirsuta* leaf development. As shown previously, *ChAS1* mRNA is detectable in leaf primordia but not in the SAM (Hay and Tsiantis 2006). We obtained higher-resolution information on *ChAS1* expression relative to developing leaflets and found that *ChAS1* transcripts accumulate in a central domain of the rachis and leaflets in *C. hirsuta* leaves (Fig. 6A–D; Supplemental Fig. S6). We observed that *ChAS1* and *ChCUC2* expression domains are near complementary during leaflet formation, while *ChBP* and *ChCUC2* domains overlap (Fig. 6E–L). These observations are consistent with the suggestion borne out of genetics that *ChAS1* and *ChCUC2* promote *C. hirsuta* leaf growth through their opposing inputs on *ChBP* across a developmental boundary that delimits leaflets (Fig. 6M). In order to refine this model, it will be important to colocalize expression of all three genes at cellular resolution. Notably, the complex interactions at the boundary of *C. hirsuta* leaflets are reminiscent of so-called paradoxical interactions underlying boundary development in animal systems (Sprinzak et al. 2010; Hart and Alon 2013) and highlight the need for further study into the cellular basis of developmental boundary function in plants (Rossmann et al. 2015).

## Discussion

Our work highlights how in-depth genetic analysis of development in a comparative context is a key tool for understanding the genetic basis for morphological diversity. We showed that two paralogous homeobox genes, *ChSTM* and *ChBP*, share redundant functions in *C. hirsuta* leaflet formation and found that *cis*-regulatory change in the less pleiotropic gene of the two, *ChBP*, had a more active role in generating diverse leaf morphologies. These comparisons provide an opportunity to visualize tradeoffs between the ability of a gene to cause morphological change and its pleiotropy and provide empirical evidence for a prediction that narrowly pleiotropic regulators and, in particular, transcription factors might be key drivers of plant diversity (Doebley and Lukens 1998).

Leaf margin morphology depends on the action of a small GRN comprising *MIR164A, CUC* genes, and auxin activity components (Supplemental Fig. S7; Blein et al. 2008; Kawamura et al. 2010; Bilsborough et al. 2011). Previous work indicated that tinkering with this GRN through alterations in KNOX activity might contribute to the evolution of divergent leaf morphologies (Ori et al. 2000; Hake and Ori 2002; Hay and Tsiantis 2006; Barkoulas et al. 2008; Blein et al. 2008; Bilsborough et al. 2011). However, the evolutionary changes underlying such diversity and their impact on the architecture of the GRN were unknown. Here we show that *cis*-regulatory divergence of *BP* provides a direct mechanistic path for creating alternate GRN architectures between simple and complex leaves. Specifically, we found that *ChBP* expression in the *C. hirsuta* leaf renders it a mediator of *mir164A/ChCUC* activity and a functionally critical target of ChAS1. This network architecture, which is not seen in *A. thaliana*, creates a species-specific interaction between *ChAS1* and *ChCUC2* that supports leaflet formation and influences leaf growth broadly. Thus, we provide an example of how regulatory evolution of a single low pleiotropy gene, *BP*, can contribute to substantial rewiring of a developmental network regulating leaf shape.

We propose that the flexible integration or disengagement of weakly pleiotropic regulators, such as *BP*, from conserved genetic networks provides a path through which regulatory evolution can alter molecular circuitries that influence organ growth. Such network rewiring may help complex organisms readily evolve morphological diversity by overcoming potential fitness penalties caused by pleiotropy (Stern and Orgogozo 2008). In the future, it will be interesting to test these possibilities by evaluating the relative pleiotropy and capacity for network reorganization of other genes that are sufficient to account for trait diversity between species. Overall, our work indicates that the interplay between pleiotropy and regulatory evolution underpins morphological change in not only metazoans, where stereotypical body plans are laid down during embryogenesis, but also seed plants, where organogenesis occurs post-embryonically and shows considerable plasticity (Steeves and Sussex 1989; Carroll et al. 2004). This interplay may therefore reflect a fundamental property of morphological evolution rather than lineage-specific constraints associated with metazoan organogenesis (Arthur 2004).

## Materials and methods

### Plant material and growth conditions

The origins of mutant alleles and transgenic lines used in this study were as follows: *chas1-1* (Hay and Tsiantis 2006), *as1-1* (CS3374, *Arabidopsis* Biological Resource Center), *bp-9* (Smith and Hake 2003), *as1-101* (Sun et al. 2002), *bp-1* (Venglat et al. 2002), *cuc2-3* (Hibara et al. 2006), *mir164a-4* (Nikovics et al. 2006), *35S::MIR164b* (Blein et al. 2008), *35S::MIR164b;35S::CUC3RNAi* (Blein et al. 2008), *35S::CUC2-GR* (Bennett et al. 2010), *35S::STM-GR* (Gallois et al. 2002), *DR5rev::VENUS* (Barkoulas et al. 2007), *PIN1::PIN1-GFP* (Heisler et al. 2005), *CUC2::CUC2-VENUS* (Heisler et al. 2005), and *BP::GUS* (Hay and Tsiantis 2006). Plants were grown on soil under long-day conditions (18 h light; 20°C). With the exception of the *chstm*/+ allele, which was maintained as a segregating pool, generation of double or triple mutants was performed by crossing of homozygous plants. These crossings were genotyped in the F2 populations and phenotyped in the F3 generation. Analysis of reporter gene expression was performed in the F2 and confirmed in the F3 and F4 generations after genetic crossing.

### Ethane methyl sulfonate (EMS) mutagenesis

For EMS mutagenesis, *C. hirsuta* wild-type (Oxford; Hay and Tsiantis 2006) or mutant seeds (*chas1-1*, *chas1-1;chstm-1*/+, or *par*) were mutagenized by agitation with 0.2% EMS (Sigma) for 10 h, washed in dH_2_O, sown on soil, and harvested in pools of five plants. M2 plants (total numbers are given below) were subsequently screened for leaf phenotypes or suppression of leaf phenotypes. Mutant characterization was performed after backcrossing to wild type at least twice. A detailed description of the *chstm-1*, *chbp-1*, *par*, and *chcuc2-1* mutant isolation and complementation is provided in the Supplemental Material.

### Binary constructs and plant transformation

All constructs were transformed into *C. hirsuta* and *A. thaliana* by floral dip (Clough and Bent 1998) using *Agrobacterium tumefaciens* strain GV3101. For a detailed description of how the constructs were generated, see the Supplemental Material. For each construct, a minimum of 15 independent transgenic lines were self-pollinated to obtain T2 seeds. The progeny of at least five independent T1 lines were analyzed in each case.

### Quantitative real time RT–PCR (qRT–PCR) analysis

The RNeasy plant minikit (Qiagen) was used for RNA extraction. Total RNA (1 µg) was treated with DNase I and transcribed into cDNA using SuperScript II reverse transcriptase and an oligo(dt) primer (Invitrogen). qRT–PCR was performed in triplicate from two independent RNA extractions using the SYBR Green PCR master mix (Applied Biosystems) and an ABI Prism 7300 sequence detection system (Applied Biosystems). The primer efficiency and expression level were determined as described (Pfaffl 2001). Expression levels were normalized to the reference gene *GLYCERALDEHYDE-3-PHOSPHATE DEHYDROGENASE* (GAPDH). With the exception of the genes *ChKNAT2* (5′-TGGCTATCTTGCGCTGCTAC-3′ and 5′-TGCAAGAGGCCTTTCAGTTT-3′) and *ChKNAT6* (5′-CGGAGATCAGAAGAAACGATGA-3′ and 5′-GCGAGGATACGATGGATGAC-3′), all primers sequences used have been published previously (Kougioumoutzi et al. 2013).

### RNA in situ hybridization

RNA in situ hybridizations on 15-µm sections through fixed and paraffin-embedded shoot apices of 2- to 3-wk-old short-day grown plants were performed as described (Vlad et al. 2014). Digoxigenin-labeled antisense RNA probes to *C. hirsuta ChAS1* were generated using cDNA templates obtained after amplification with the primer combinations 5′-AGTAGTGAGAGTGTGTTCTTGTC-3′ and 5′-CCAAGCTTCTAATACGACTCACTATAGGGAGATCTAATCTGCAACCCATG-3′ (the T7 RNA polymerase-binding motif is underlined). To cover the entire hybridization pattern, several consecutive sections were registered, and minimal projections were generated using the image processing package Fijii (Supplemental Fig. S6; Schindelin et al. 2012). The signal was observed and images were acquired with a Zeiss Axiophot light microscope and a Leica DFC 490 digital camera.

### Phenotypic analysis and estimation of pleiotropy

Quantification of lateral, intercalary, axillary, or secondary leaflets was done with at least 15 individual wild-type or mutant plants, and each experiment was repeated at least twice. To obtain leaf silhouettes, fully developed leaves were flattened onto clear adhesive on white paper and then digitally scanned. Leaf length, area, and perimeter were calculated from silhouettes using Fijii software (Schindelin et al. 2012). The petiole cell length and number was measured as described (Rast and Simon 2012; Vlad et al. 2014).

The degree of reduction of plant rosette diameter and, for selected genotypes, the total number of intact ovules per silique were used as pleiotropy estimates. Plant rosette diameter was calculated from photographs using Fiji software (*n* ≥ 15). Siliques 6–10 of the first side shoot (*n* ≥ 45) of at least 10 independent plants were collected, and ovules were counted. An average number of ovules per silique was then calculated from this sample. Both rosette diameter and seed number were then plotted as 1/*x* against the leaf dissection index (perimeter squared)/(4π × area) (Bilsborough et al. 2011).

### Scanning electron microscopy (SEM), confocal laser scanning microscopy, and light microscopy

SEM and confocal laser-scanning microscopy were carried out as described (Bilsborough et al. 2011). SEM samples were analyzed using a JSM-5510 microscope (Jeol). For the analysis of fluorescence reporter expression, seedlings were mounted and observed in water without fixation. Confocal imaging was performed using a Leica TCS SP5 II microscope and a 10× objective (HC PL Fluotar 10 9 0.30) or a water dipping 20× objective (HCX APOLU-V-I 0 9 0.5) or a Zeiss LSM 780 upright microscope and water immersion objective (AP 20×/0.8 M27). Visualization of VENUS expression was performed using a 488-nm argon laser and a 657- to 743-nm filter for the chlorophyll autofluorescence and a 505- to 550-nm bandpass filter. Maximal projections were generated from stacks of five to 30 sections. GUS activity was detected as described in Hay and Tsiantis (2006). Imaging of GUS-stained samples and agarose prints was performed using a Leica DFC 490 digital camera mounted on a Zeiss Axiophot light microscope. Images were processed and analyzed using Fijii and Adobe Photoshop software.

## Supplementary Material

Supplemental Material
