# Supplementary material for: Alternate wiring of a KNOXI genetic network underlies differences in leaf development of A. thaliana and C. hirsuta
Source: Genes Dev. 2015 Nov 15;29(22):2391–404. doi: 10.1101/gad.269050.115 (PMC4691893; doi:10.1101/gad.269050.115)
Supplement: Supplemental Material [file supp_29_22_2391__index.html]

Supplemental Material 

# Alternate wiring of a *KNOXI* genetic network underlies differences in leaf development of *A. thaliana* and *C. hirsuta*

## Supplemental Material

**Files in this Data Supplement:**

- Supplemental Information.pdf
