# Supplementary material for: Alternate wiring of a KNOXI genetic network underlies differences in leaf development of A. thaliana and C. hirsuta
Source: Genes Dev. 2015 Nov 15;29(22):2391–404. doi: 10.1101/gad.269050.115 (PMC4691893; doi:10.1101/gad.269050.115)
Supplement: Supplemental Material [file supp_29.22.2391_Supplemental_Information.pdf]

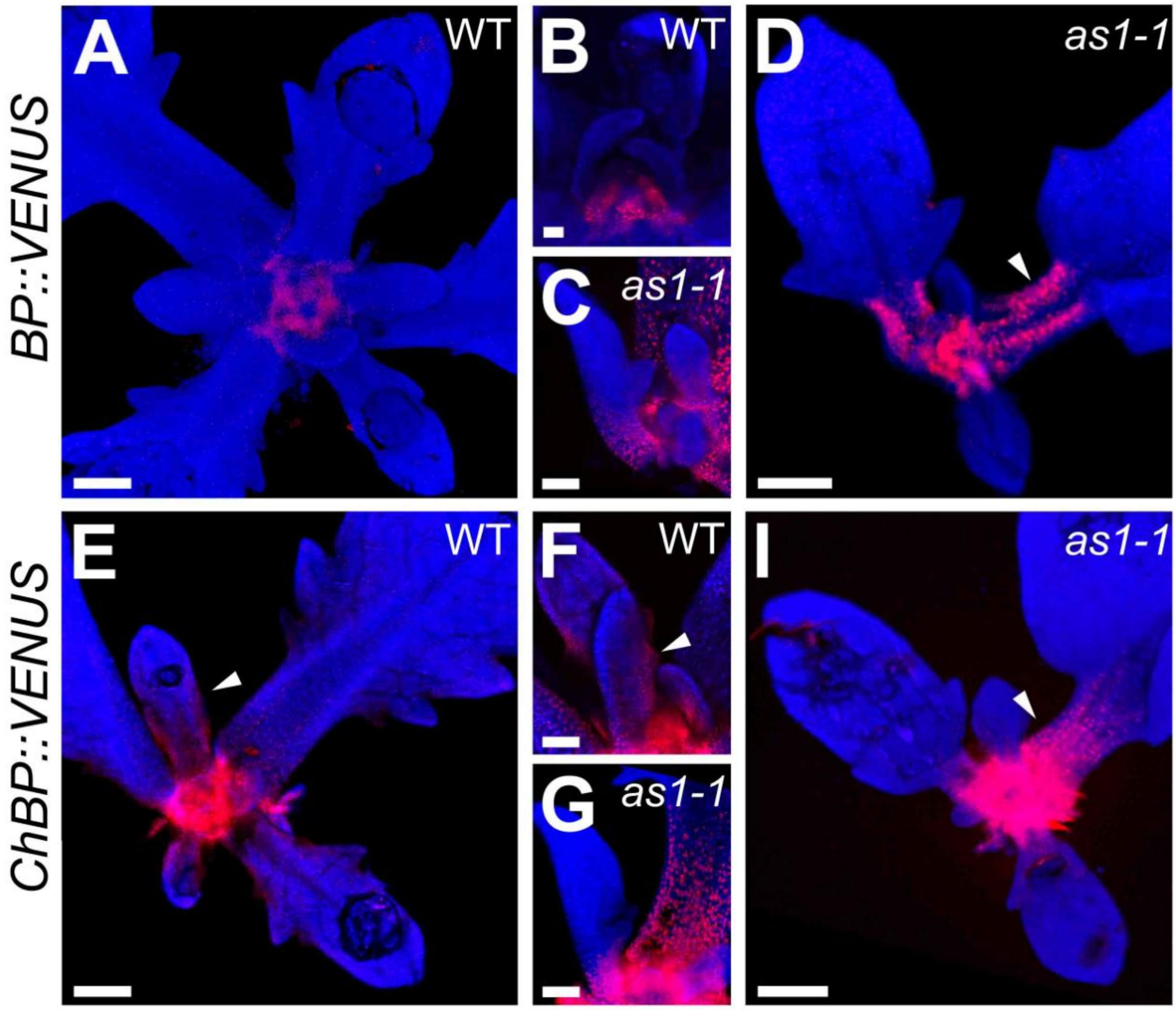

Figure S1: *cis*-regulatory diversification causes species-specific *BP* expression. (A-I) Maximum intensity projections of confocal stacks showing (A-D) *BP::3xVENUS* and (E-I) *ChBP::3xVENUS* expression (red) combined with chlorophyll autofluorescence (blue) in *A. thaliana* wild-type (A-B, E-F) and *as1-1* mutants (C-D, G-I). Note that the *ChBP* promoter drives expression in *A. thaliana* wild-type leaves (E-F, arrowhead), while expression from the endogenous *BP* promoter is restricted to the SAM (A-B). Both *BP::3xVENUS* and *ChBP::3xVENUS* show broadened expression in *as1-1* mutants of *A. thaliana* (C-D, G-I, arrowhead) relative to the respective wild type. Scale bars (A, D, E, I): 1 cm; (B, C, F, G): 100 µm.

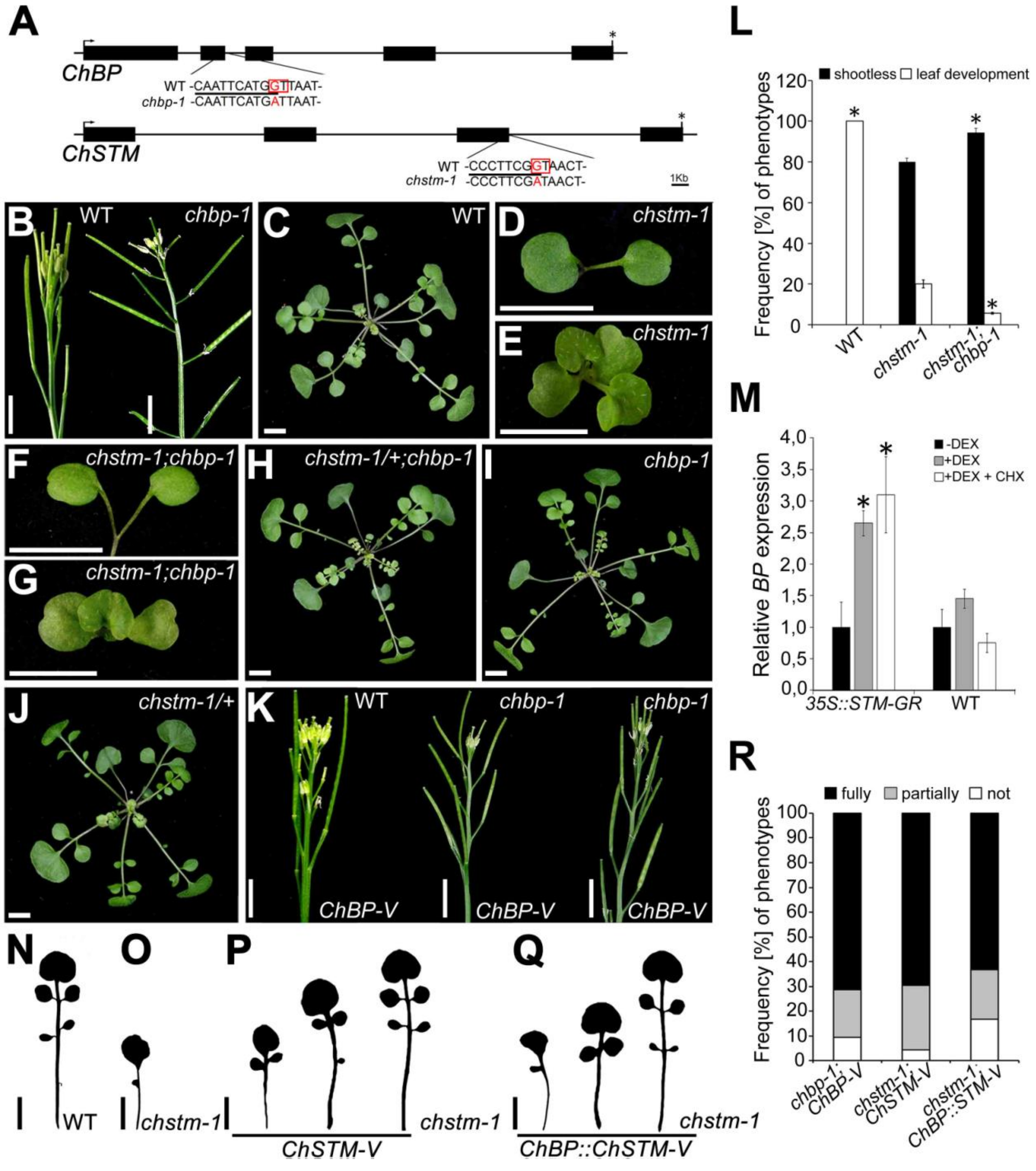

Figure S2: Isolation and characterization of the *C. hirsuta chbp-1* and *chstm* single and double mutants. (A) Gene structure of *ChBP* and *ChSTM.* The position of the point mutations (G to A) is indicated. Black boxes: exons; arrows: ATG; asterisk: STOP codon; red letter: point mutation; red box: invariant splice site at the end of *ChBP* exon2 and *ChSTM* exon3; underlined: exon sequence. **(B)** Inflorescence of *C. hirsuta* WT and *chbp-1* mutant. **(C-J)**

Rosettes of *C. hirsuta* WT (C), *chstm-1* (D-E), *chstm-1; chbp-1* (F-G); *chstm-1/+; chbp-1* (H), *chbp-1* (I) and *chstm-1/+* (J) mutant plants at 20 DAG (C, D, F, H - I) and 28 DAG (E, G). The majority of *chstm-1* and *chstm-1;chbp-1* remains shootless (D, F) but a few recover to develop 1-5 leaves (E, G)**. (K)** Inflorescence of wildtype plants expressing the *ChBP-V* transgene and partially complemented and fully complemented *chbp-1; ChBP-V* plants (left to right). **(L)** Frequency of *chstm-1* or *chstm-1;chbp-1* mutants (28DAG) that develop leaves (white bars) or remained shootless (black bars). **(M)** Induction of *STM* with 10mM DEX in *35S::STM-GR A. thaliana* plants results in an upregulation of *BP* expression within 2 hours*,* even in the presence of the protein synthesis inhibitor CHX (30 mM) indicating a direct regulation (n = 3). **(N-Q)** Leaf 5 silhouettes of *C. hirsuta* WT (N), *chstm-1* mutants (O), *chstm-1; ChSTM::ChSTM-VENUS* (P) and *chstm-1; ChBP::ChSTM-VENUS* (Q) plants. (P-Q) from left to right: not, partially and fully complemented plants. **(R)** Frequencies of not, partially and fully complemented T1 plants (n ≥ 45). Scale bars: 1cm; Error bars (L-M): Standard deviation; Asterisk: Statistically significant difference to *chstm* (L) or uninduced plants (M) (Student's *t*-test; p>0.05).

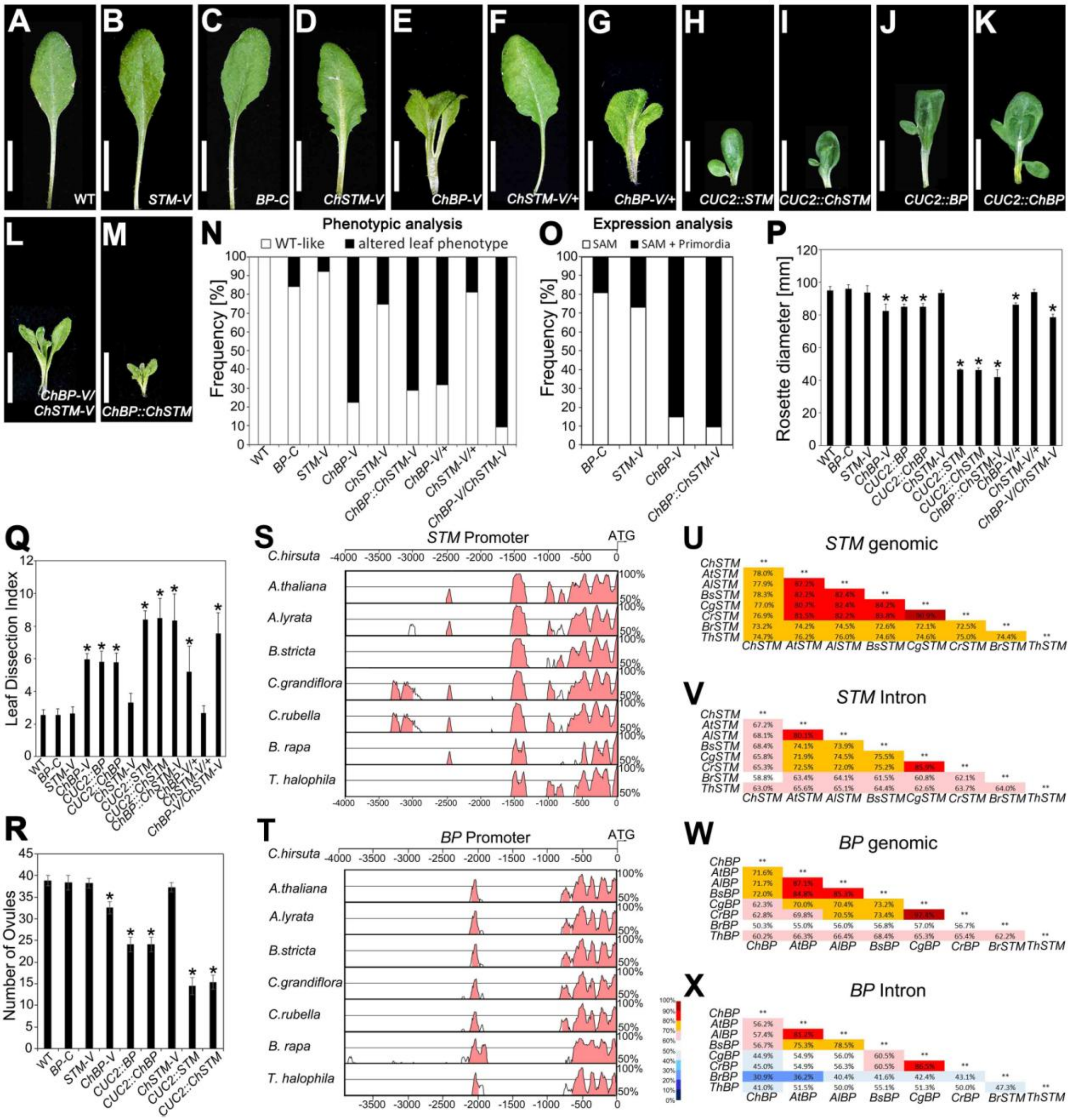

Figure S3: The effect of transgenic *BP/ChBP* and *STM/ChSTM* expression on *A. thaliana* development. (A-M) Fifth rosette leaf 5 of *A. thaliana* WT (A), and *A. thaliana* plants expressing the indicated transgenes (B to M). **(N)** Frequency of altered leaf phenotypes (black bars) observed in the different single transgenic lines (n ≥ 75; T2 plants of n ≥ 10 independent lines). Double transgenic lines were analyzed in the F1 (n ≥ 35 F1 plants respectively). Crossing of *ChBP::ChBP-VENUS* (*ChBP-V/+*) and *ChSTM::ChSTM-VENUS* (*ChSTM-V/+*) to wild-type was performed to control for the effect of

transgenic zygosity. **(O)** Frequency of T2 plants showing transgene expression only in the SAM (white bars) or in the SAM and leaf primordia (black bars). **(P)** Quantification of rosette diameter [mm] in *A. thaliana* and *C. hirsuta* WT compared to different transgenic lines ($n \geq 15$). **(Q)** Quantification of the leaf dissection index for leaf 5 of *A. thaliana* and *C. hirsuta* WT compared to different transgenic *A. thaliana* lines ($n \geq 15$, (Bilsborough et al. 2011)) **(R)** Quantification of ovule number per silique in *A. thaliana* WT compared to different transgenic lines ($N \geq 50$). **(S-T)** VISTA analysis (http://genome.lbl.gov/vista/index.shtml) of all promoter sequences in Y axis to the promoter of *C. hirsuta ChSTM* (S) and *ChBP* (T) revealed the evolutionarily conserved (coloured boxes) *cis*-regulatory regions. Pairwise alignments were done by Shuffle-LAGAN (70% identity threshold and 100 bp sliding window) and visualized with VISTA Browser. Red colors (pink peaks) depict the regions, which have at least 70% identity with the *ChSTM* or *ChBP* promoter. The translation start site for each gene is identified by an arrow. **(U-X)** Matrices of pairwise identity percentages of the *STM* and *BP* genomic regions (U, W; ATG to STOP) and intragenic regions (V-X) from *C.hirsuta, A.thaliana, A.lyrata, B.stricta, C.grandiflora, C.rubella, B.rapa* and *T.halophila.* The matrices were generated using the full optimal alignment method and the observed divergency distance method options of MUSCLE software. Percentages of identity are indicated with a colour code (see legend). Scale bars (A-M): 1 cm, Error bars (P-R): Standard deviation, Asterisk (P-R): Statistically significant difference to *A. thaliana* WT (Student's *t*-test; $p > 0.05$).

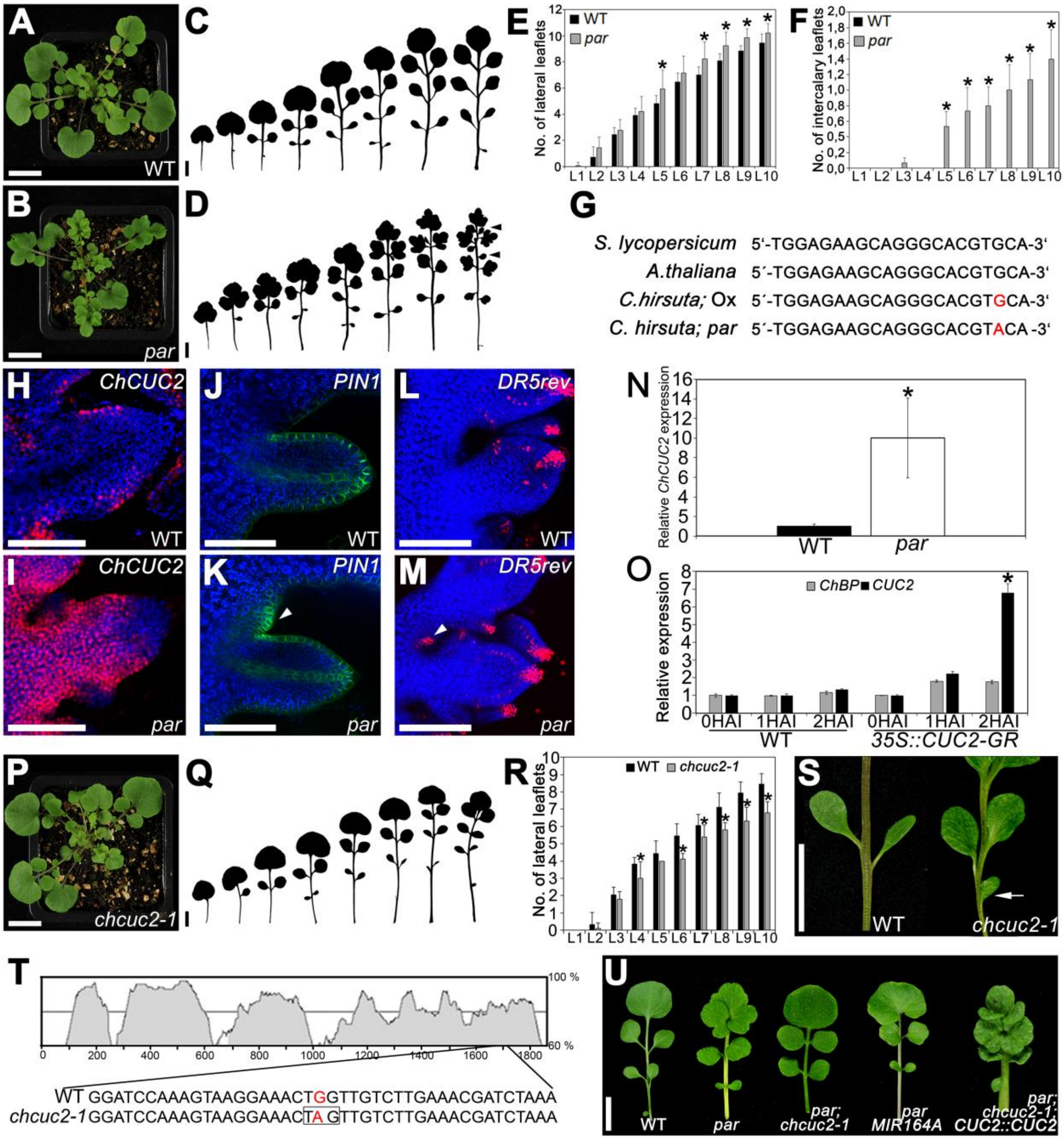

Figure S4: Characterization and isolation of the *C. hirsuta par* and *chcuc2-1* mutant. (A-D) Rosettes (A-B) and heteroblastic leaf series (C-D) of *C. hirsuta* WT (A,C) and *par* (B,D) mutants. **(E-F)** Quantification of lateral leaflet number (E) and intercalary leaflet number (F) in rosette leaves 1-8 of *C. hirsuta* WT and *par* mutants (n ≥ 15). **(G)** Sequencing of *par* identifies a G to A point mutation in the 19th nucleotide of the 21bp microRNA *miR164*, which is fully conserved between *A. thaliana*, *S. lycopersum* and *C. hirsuta.* **(H-M)** Maximal

projections of confocal stacks showing *ChCUC2g-VENUS* (J-K, red), *PIN1::PIN1-GFP* (L-M, green) and *DR5rev::VENUS* (N-O, red) reporter gene expression combined with chlorophyll autofluorescence (blue) in lateral leaflets of *C. hirsuta* WT and *par* mutant leaf 5 (750 µm). **(N)** Relative expression of *ChCUC2* in WT and *par* mutants (n = 3). **(O)** Relative expression of *ChBP* and *CUC2* in *C. hirsuta* WT and *35S::CUC2-GR* plans upon 0, 1, 2 and 12 hours after induction (HAI) with 10 µM DEX (n = 3). **(P-Q)** Rosette (P) and heteroblastic leaf series (Q) of *C. hirsuta chcuc2-1* mutants. **(R)** Quantification of lateral leaflet number in rosette leaves 1-10 of *C. hirsuta* WT and *chcuc2-1* mutants (n ≥ 15). **(S)** Close up of leaf 5 rachis of WT and *chcuc2-1* mutants. 9.5 % of *chcuc2-1* rosette leaves showed minor fusion of the petioule to the rachis (n = 111). **(T)** Vista alignment of *A. thaliana* and *C. hirsuta CUC2* open reading frame. The position of a point mutation in the *chcuc2-1* mutant (G to A, red letters) is indicated. **(U)** Leaf 5 of *C. hirsuta* WT, *par; par*; *MIR164A::MIR164A* (*MIR164A*) complemented mutants; *par;chcuc2-1* and transgenic *par;chcuc2-1;CUC2::CUC2-VENUS* plants. The *CUC2::CUC2-VENUS* transgene complements the loss of *ChCUC2* function in *par;chcuc2-1* mutants. Scale bars (A-D, P-Q, U): 1cm; (J-O): 100 µm; Error bars: Standard deviation; Asterisk: Statistically significant difference to WT or uninduced plants (Student's *t*-test; p≤0.05). Arrowheads: intercalary leaflets.

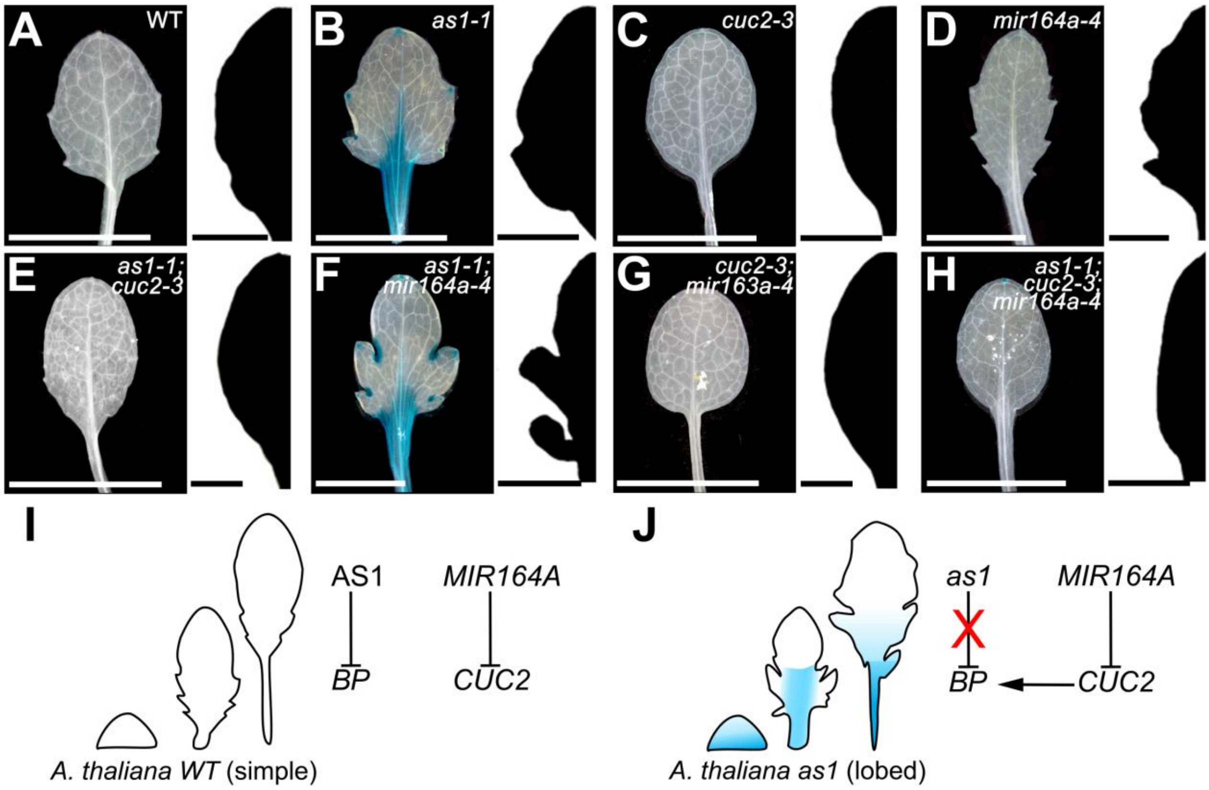

Figure S5: *CUC2* is required for *BP* misexpression in *A. thaliana as1-1* mutant leaves. (A-H) Rosette leaf 5 stained for *BP::GUS* (blue) and half silhouette of *A. thaliana* WT (A), *as1-1* (B), *cuc2-3* (C), *mir164a-4* (D), *as1-1;cuc2-3* (E), *as1-1;mir164a-4* (F)*; cuc2-3;mir164a-4* (G) and *as1-1;cuc2-3;mir164a-4* (H) mutants*.* The *as1-1* leaf phenotype and ectopic *BP* expression is enhanced by the *mir164a-4* mutation (B, F). This enhancement is *CUC2* dependent as the *BP::GUS* reporter gene expression is confined to the SAM in *as1-1;cuc2-3* (E) and *as1-1;cuc2-3;mir164a-4* (H) mutants. **(I-J)** Schematic representations showing, from left to right, a leaf primordia, a 500 µm leaf and a mature leaf in *A. thaliana* WT (I) and *as1* (J) mutant. (I) AS1 restricts *BP* expression from the leaf where *MIR164A* restricts *CUC2* expression. (J) *BP* misexpression in the *A. thaliana* leaf, for instance in *as1* mutants, brings *BP* under a MIR164A /CUC2 dependent regulation. Scale bars: 1 cm; Blue: *BP* expression.

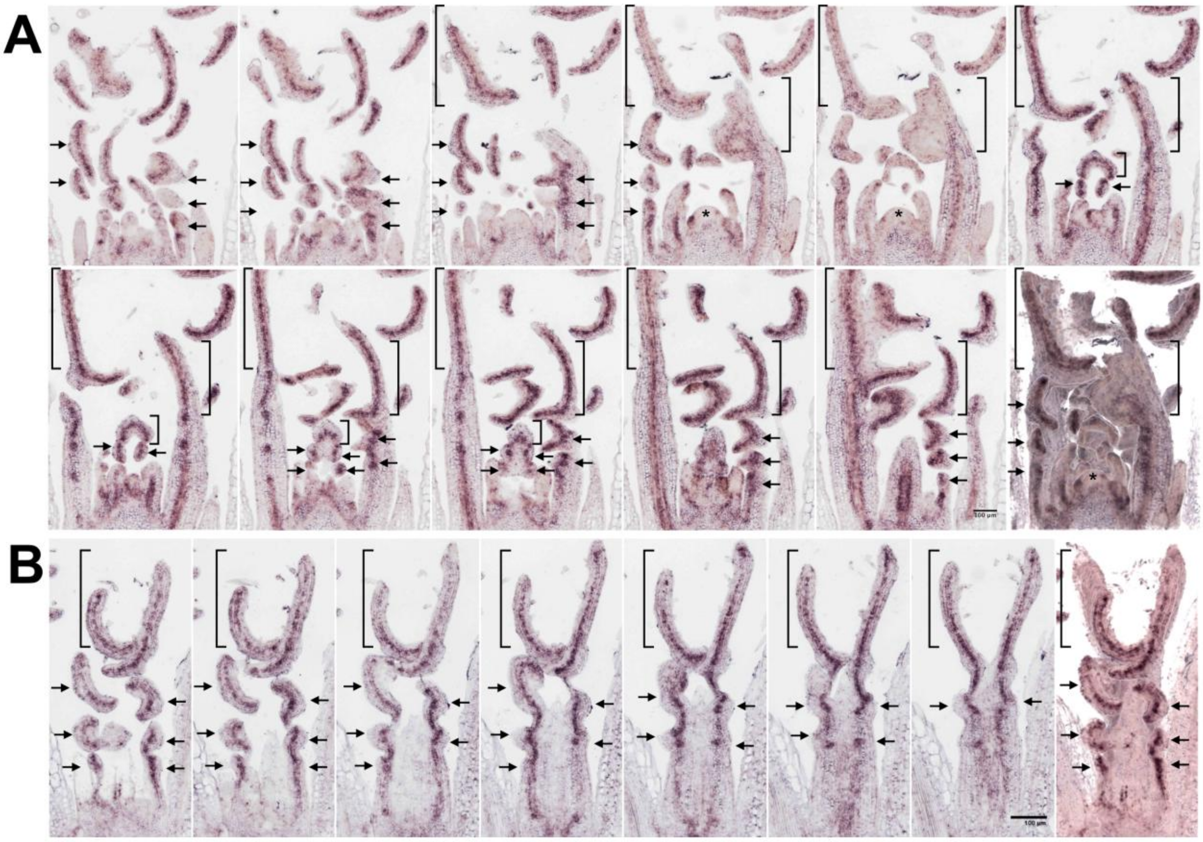

Figure S6: Expression pattern of *ChAS1* during *C. hirsuta* leaf development. (A-B) RNA *in situ* hybridizations were performed on longitudinal sections through the apical region of a vegetative shoot (A) and a 750µm leaf (B) of *C. hirsuta*. Minimum projections and corresponding consecutive sections are shown. *ChAS1* expression is absent from the shoot apical meristem (asterisk, A) but detectable in incipient early organ primordia. Later, expression appears in a central region of the leaf rachis, lateral leaflets (arrows A, B) and terminal leaflets (brackets A, B). A close up of (A) showing a lateral leaflet and one of the sections shown in (B) are also presented in Fig. 6C, D and are repeated here for clarity. Scale bars: 100 µm

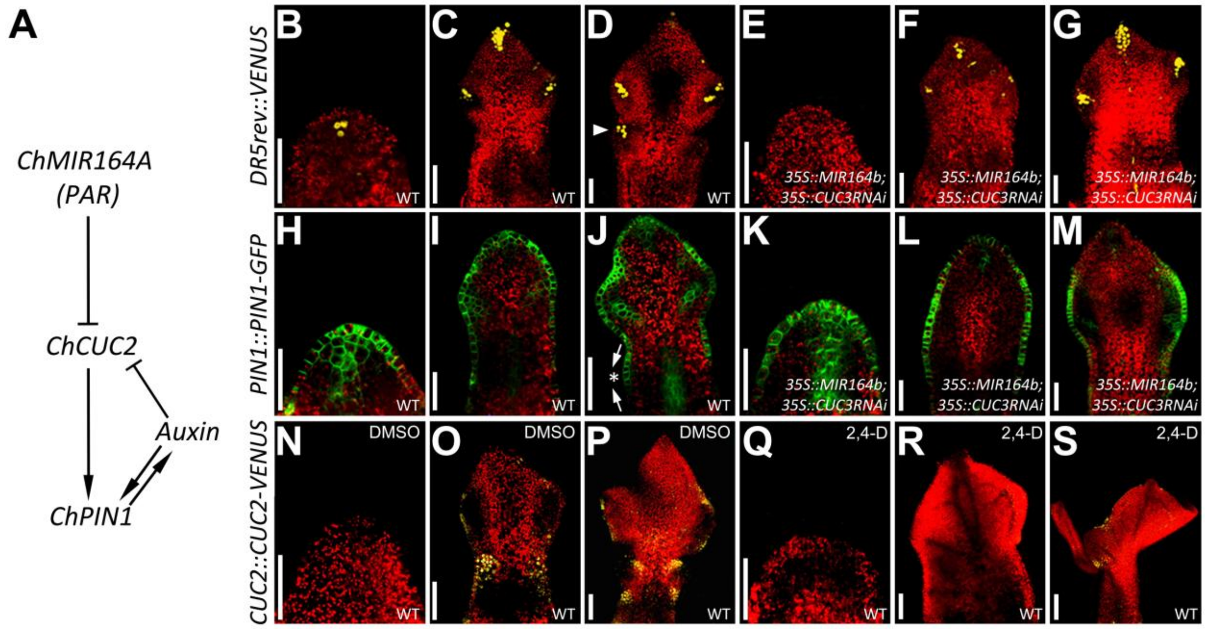

Figure S7: *ChCUC* function is required for the formation of auxin activity maxima in *C. hirsuta* leaves. (A) Schematic representation of the genetic interactions shown here or in previous studies. **(B-S)** Maximal projections of confocal stacks showing *DR5rev::VENUS* (B-G, yellow), *PIN1::PIN1-GFP* (H-M, green) and *CUC2::CUC2-VENUS* (N-S, yellow) reporter gene expression combined with chlorophyll autofluorescence (red) in *C. hirsuta* leaf 5 at three different stages of development [100 µm, 200 µm and 400 µm leaf length]. (B-M) Auxin activity maxima (insets in D, G) and PIN1 convergence points (insets in J, M) are not formed along the leaf rachis of *35S::MIR164b; 35S::CUC3RNAi* transgenic lines but are visible in WT leaves (arrowhead in D, asterisk in J). (N-S) External treatments with 10µM 2,4-D (Q-S) or with DMSO as control (N-P) reveals that auxin likely represses *ChCUC2* expression during early stages of *C. hirsuta* leaf development. Scale bars: 50 µm

SUPPLEMENTAL MATERIAL AND METHODS

Isolation, mapping and complementation of *C. hirsuta* EMS mutants

The *chstm-1* mutant was isolated from EMS-treated wild type seeds by visual inspection of ~1,500 EMS M2 plants (Hay and Tsiantis 2006). PCR amplification and sequencing of the *ChSTM* locus of *chstm-1* mutants led to the identification of a G/A transition at a GT invariant splice site. This results in a failure to splice intron 3 and introduced a premature STOP codon in the reading frame of the mutant. For genotyping, a CAPS marker was designed by EcoRV digestion of the ChSTM-CAPSF/R PCR product (5'-ATCCGCAAGCTGAGGATAGA-3' and 5'-GTTATTTAGCGAGAGTTTAGAAGAATAAAGATA-3'). The introduction of the *ChSTM::ChSTM-VENUS* (*ChSTM-V*) transgene fully complemented the phenotype in 24 and partially complemented the phenotype in 21 out of 47 independent *chstm-1* T1 lines (Fig. S2R).

The *chbp-1* allele was isolated from EMS-mutagenized *chas1-1;chstm-1/+* seeds by visual inspection of ~20,000 EMS M2 plants. We reasoned that screening of *chas1-1;chstm-1/+* would help us distinguish *KNOXI*-dependent and independent *ChAS1* targets potentially obtained from the screen. Genomic DNA and cDNA of wild type and *chas1-1;chbp-1* was isolated, amplified and sequenced to assemble the contigs. This resulted in the identification of a G/A point mutation that created a premature stop codon in the second intron of the *ChBP* locus. A CAPS marker for genotyping was designed by *Bsp*HI digestion of the cBP-CAPS-F/R PCR product (5'-CCCACCTGAAGTGGTTGATAA-3' and 5'-ATACACAAAGGCCCAACTCC-3'). The *chbp-1* single mutant was isolated by back crossing *chas1-1;chbp-1* several times to wild type. Introduction of a *ChBP::ChBP-VENUS* (*ChBP-V*) transgene fully complemented the downward pointing silique phenotype of *cbp-1* mutants in 34 out of 48 independent *chbp-1* T1 lines and partially complemented the phenotype in 12 T1 lines (Fig. S2R).

The *par* mutant was isolated from EMS-treated wild type seeds by visual inspection of ~1,500 EMS M2 plants (Hay and Tsiantis 2006). A F2 mapping population was generated by out-crossing *par* to a polymorphic Greek strain. For map-based cloning, genomic DNA was isolated from 131 *par* mutants segregating in a 3:1 Mendelian ratio. A first rough mapping linked the *par* mutant phenotype to a 120kb region syntenic to the southern tip of chromosome 2 of *A. thaliana* containing 32 predicted genes. For fine mapping, new CAPS, dCAPS or length markers were designed based upon sequence differences between BAC or *C. hirstua* WT sequences and the Greek ecotype. Primers used to subsequently amplify and sequence the *ChMIR164A* locus were designed based upon the synteny in nucleotide sequence between *C. hirsuta*, *A. thaliana* and *Brassica rapa*. Following the identification of a G/A point mutation in the 19th nucleotide of the conserved 21bp *miRNA164* in *par*, an RsaI CAPS marker for genotyping was designed (5'-TCCATTAATGCAGCCTTTGG-3' and 5'-GGAGCTCATGTTGGAGAAGG-3'). Complementation of 10 out of 12 independent T1 *C. hirsuta* lines with a *MIR164A::MIR164A* transgene (Nikovics et al. 2006) confirmed loss of *ChMIR164A* function in *par* (Fig. S4U).

The *chcuc2-1* allele was isolated from EMS-treated *par* seeds by visually screening ~9,000 M2 EMS plants for suppression of the *par* leaf phenotype. The suppressed lines were genotyped for *par* using the *Rsa*I CAPS marker and the *ChMIR164A* locus was sequenced to exclude an intragenic suppressor. The *ChCUC2* locus of wild type and *par;chcuc2-1* mutants was then amplified and sequenced. Following identification of a G/A point mutation in the *ChCUC2* locus of *par;chcuc2-1,* a *Spe*I CAPS marker was designed (5'-CAAACAGACTTGGGCTTAGACG-3' and 5'-AACGGAGGAAGTCATTGTGG-3'). The *chcuc2-1* single mutant was eventually isolated by outcrossing *par;chcuc2-1* to wild type. A *CUC2::CUC2*-

VENUS transgene (Heisler et al. 2005) was sufficient to complement *chcuc2* in 6 out of 9 T2 *par;chcuc2-1* lines, causing a reversion back to the *par* phenotype (Fig. S4U).

Binary Constructs and Plant Transformation

To construct a *C.hirsuta ChBP::VENUS* reporter, the upstream regulatory sequences of *ChBP* were amplified using the primer 5'-GAATTCTTTAACATCCCGA-3' and 5'-ACCCAGATGAGTAATATTTG-3' (3. 982 Kb). The PCR product was subcloned as a SalI/XhoI fragment in pBJ97 (Gleave 1992), upstream of the 3xVENUS sequence previously inserted into a SpeI-blunted site (pBJ97-VENUS). The whole cassette was cloned as a NotI fragment into pMLBART (Gleave 1992).

For the *C.hirsuta ChBP::ChBP-VENUS* construct, the *ChBP* ORF was amplified from genomic DNA using the primer 5'-CCGGTACCATGGAAGAATATCAGCACAACAC-3' and 5'-CGCAGCCATGGCTGGGCCAAGACGATAAGGTCC-3'. This fragment was subcloned into the BamHI/NcoI restriction sites of the intermediate pBJ97-VENUS vector. The *ChBP* promoter was excised from the *ChBP::VENUS* - pBJ97 vector described previously and inserted into a blunted BamHI restriction site. The entire cassette was subsequently moved as NotI fragment to pMLBART.

The *BP::BP-CFP* transgene was generated by PCR amplification of a 5825 bp genomic fragment from the *BP* locus using primers (5-CTCGAGTGAAAATCGCCAAAATGTCAATACA-3) and (5-GGATCCGCTGGACCGAGACGATAAGGTCCATC-3) and cloning as a translational fusion upstream of a 9 Ala linker followed two tandem copies of eCFP (Clontech Laboratories, Inc.) and OCS 3' terminator sequences. The 8.1 kb transgene was excised with *Not*I and cloned into the binary vector pMLBART. This fusion protein appears to function to complement the *bp-1* mutant.

To make the *BP::VENUS* reporter, the *BP* promoter was cloned as a BamHI fragment into the intermediate pBJ97-Venus Vector. This *BP::VENUS* cassette was then inserted into the NotI restriction site of pMLBART.

To make *MIR164A::ChBP*, the *MIR164A* promoter (1.9 Kb) was amplified from the *MIR164A::MIR164A* (Nikovics et al. 2006) using the primers 5'-GCTGATGACGTCAGCGATAATTGGGGTC-3' and 5'-ACGTGAAGATTCTCACCCCGCATTTCC-3' and sub-cloned into the vector pBJ36 (Eshed et al. 2001). A blunted *ChBP* cDNA fragment was then cloned in the SmaI site of pBJ36 and the cassette was moved to pMLBART as a NotI fragment.

For the *CUC2::ChBP* and *CUC2::BP* constructs, the *A. thaliana CUC2* promoter (3.142 Kb) was amplified with flanking *Nsi*I/*Sal*I restriction sites using the primers 5'-GAGAGAATGCATTAGAGGAAGAGTTAAGAGATGAAGAAGAAAG-3' and 5'-TCTCTCGTCGACTAAGAAGAAAGATCTAAAGCTTTTGTTTGAGAGAG -3' and cloned into intermediate vector pBJ36 (pBJ36-CUC2). The *ChBP* (5'-TAGTTGGAATAGGTTCATGGAAGAATATCAGCACAACACCAG-3' and 5'-AGTATGGAGTTGGGTTCCTATGGGCCAAGACGATAAGGTC-3') and *BP* (5'-TAGTTGGAATAGGTTCATGGAAGAATACCAGCATGACAACAGC-3' and 5'-AGTATGGAGTTGGGTTCTTATGGACCGAGACGATAAGGTCCATC-3') coding sequences were amplified from cDNA pools and cloned into pPLV25. Subsequently, the CDSs were subcloned as *Xho*I/*Xma*I fragments into pBJ36-CUC2 and the whole expression cassette was transferred into the binary vector pMLBART using the NotI sites.

<u>Binary *C. hirsuta* and *A.thaliana ChSTM/STM* constructs</u>

To make the *C. hirsuta ChSTM::ChSTM-VENUS* construct, the *ChSTM* promoter (4Kb) was PCR amplified from a genomic clone (Hay and Tsiantis 2006) using the primers 5'-

GTAAAACGACGGCCAGT-3’ and 5’-CGGGATCCCTTCTCTTTCTCTCACTAG-3’ and cloned into pGem-T-Easy (Promega). This promoter fragment was then subcloned into the SalI/BamHI restriction sites of pBJ36. The gene ORF fused to VENUS was synthesized with BamHI restriction sites and subcloned into pUC57 (GenScript; Hong Kong Ltd). This fragment was then inserted as a BamHI fragment into pBJ36 containing the *ChSTM* promoter and eventually moved as a NotI fragment into pMLBART. The *A. thaliana STM::STM-VENUS* construct used in this study was previously published (Heisler et al. 2005).

To make a *ChBP::ChSTM-VENUS* construct, the synthesized *ChSTM-VENUS* fragment previously described was subcloned into the BamHI restriction sites of pBJ36. The *ChBP* upstream regulatory region was inserted as a SalI/XhoI fragment from the intermediate ChBPp-pBJ97 vector (see above) and the entire cassette was moved to pMLBART as a NotI fragment.

For the expression of *ChSTM* and *STM* from the *CUC2* promoter, the *ChSTM* (5’-TAGTTGGAATAGGTTCATGGAGAGTGGTTCCAACAGCAC-3’ and 5’-AGTATGGAGTTGGGTTCTCAAAGCATGGTTGAGGAGATGTGATC-3’) and *STM* (5’-TAGTTGGAATAGGTTCATGGAGAGTGGTTCCAACAGCAC-3’ and 5’-AGTATGGAGTTGGGTTCTCAAAGCATGGTGGAGGAGATGTG-3’) coding sequences were amplified from cDNA pools and cloned into pPLV25. Both CDSs were then subcloned as *Xho*I/*Xma*I fragments into pBJ36-CUC2 (described above) and the whole expression cassette was transferred into binary vector pMLBART using the NotI sites.

A two component trans-activation system was used for misexpression of *ChSTM* in *C.hirsuta*. The *ChSTM-VENUS* fragment with flanking BamHI sites was cloned into pVTOP and transformed into a 35S:LhGR2 driver line (provided by I. Moore, 35S::LhGR>>STM-VENUS (Craft et al. 2005)).

Binary *C. hirsuta ChCUC2* constructs

The *ChCUC2* locus was amplified in 2 PCR reactions from a BAC clone. The primers used are 5'-TCCAAGCTTTTACTCCATGGTT-3' and 5'-CAGCCATGGCGTAGTTCCAGATGCAATCAA-3' for the amplification of 750bp of the *ChCUC2* coding region with NcoI restriction sites, and 5'-GGATCCTACAGATTTTAAAGAATTCG-3' and 5'-TGCAGAAGAAGAGGATCCAAA-3' for the amplification of a 5.35Kb fragment containing the *ChCUC2* upstream regulatory sequences and BamHI restriction sites. The 750bp PCR product was then first cloned into the NcoI restriction sites of the pBJ97-3xVENUS intermediate vector described above, followed by a insertion of the 5.35Kb PCR fragment using the BamHI restriction sites. The entire cassette was then cloned as a NotI fragment into the binary vector pMLBART.

35S::amirKNT2/6 (ckn2/6)

This construct allows expression of an artificial microRNA directed against *KNAT2* and *KNAT6* (*amirKNT2/6:* TAGTTCGTCGGATCCAGACAA) from the 35S promoter. To simplify matters we refer to *35S::amirKNT2/6* as *amirKNT2/6* throughout the manuscript. The *amirKNT2/6* sequence was obtained by targeted mutagenesis of *mir319a* (pRS300, wmd3.weigelworld.org) utilizing the following primers: 1amirknat2,6I: 5'-gaTAGTTCGTCGGATCCAGACAAtctctcttttgtattcc-3', 1amirknat2,6II: 5'-gaTTGTCTGGATCCGACGAACTAtcaaagagaatcaatga-3', 1amirknat2,6III: 5'-gaTTATCTGGATCCGTCGAACTTtcacaggtcgtgatatg-3'; 1amirknat2,6IV: 5'-gaAAGTTCGACGGATCCAGATAAtctacatatatattcct-3'; A: 5'- CTGCAAGGCGATTAAGTTGGGTAAC -3' and B: 5'-GCGGATAACAATTTCACACAGGAAACAG -3'. The obtained *amirKNT2/6* was first cloned in pGemT-easy (Promega©), sequenced and then inserted as a XhoI/Xba fragment into the pART7 vector. The expression cassette was then cloned into the NotI restriction site of pMLBART.

SUPPLEMENTAL REFERENCES

Bilsborough GD, Runions A, Barkoulas M, Jenkins HW, Hasson A, Galinha C, Laufs P, Hay A, Prusinkiewicz P, Tsiantis M. 2011. Model for the regulation of Arabidopsis thaliana leaf margin development. *Proceedings of the National Academy of Sciences of the United States of America* **108**: 3424-3429.

Craft J, Samalova M, Baroux C, Townley H, Martinez A, Jepson I, Tsiantis M, Moore I. 2005. New pOp/LhG4 vectors for stringent glucocorticoid-dependent transgene expression in Arabidopsis. *The Plant journal : for cell and molecular biology* **41**: 899-918.

Eshed Y, Baum SF, Perea JV, Bowman JL. 2001. Establishment of polarity in lateral organs of plants. *Current biology : CB* **11**: 1251-1260.

Gleave AP. 1992. A versatile binary vector system with a T-DNA organisational structure conducive to efficient integration of cloned DNA into the plant genome. *Plant Mol Biol* **20**: 1203-1207.

Hay A, Tsiantis M. 2006. The genetic basis for differences in leaf form between Arabidopsis thaliana and its wild relative Cardamine hirsuta. *Nature genetics* **38**: 942-947.

Heisler MG, Ohno C, Das P, Sieber P, Reddy GV, Long JA, Meyerowitz EM. 2005. Patterns of auxin transport and gene expression during primordium development revealed by live imaging of the Arabidopsis inflorescence meristem. *Current biology : CB* **15**: 1899-1911.

Nikovics K, Blein T, Peaucelle A, Ishida T, Morin H, Aida M, Laufs P. 2006. The balance between the MIR164A and CUC2 genes controls leaf margin serration in Arabidopsis. *The Plant cell* **18**: 2929-2945.
